# Evidence update on the cancer risk of vaping e-cigarettes: A systematic review

**DOI:** 10.18332/tid/192934

**Published:** 2025-01-28

**Authors:** Anasua Kundu, Kyran Sachdeva, Anna Feore, Sherald Sanchez, Megan Sutton, Siddharth Seth, Robert Schwartz, Michael Chaiton

**Affiliations:** 1 Institute of Medical Science, University of Toronto, Toronto, Canada; 2 Faculty of Health Sciences, Queen’s University, Kingston, Canada; 3 School of Medicine, University College Cork, Cork, Ireland; 4 Faculty of Medical Sciences, Western University, Ontario, Canada; 5 Centre for Addiction and Mental Health, Toronto, Canada; 6 Dalla Lana School of Public Health, University of Toronto, Toronto, Canada

**Keywords:** e-cigarette, vaping, cancer, lung cancer, neoplasm

## Abstract

**INTRODUCTION:**

There is substantial interest in the association of vaping e-cigarettes with the risk of cancer. We analyzed this risk in different populations by updating the Kings College London (KCL) review to include the period between July 2021 and December 2023.

**METHODS:**

We searched six databases and included peer-reviewed human, animal, and cell/*in vitro* original studies examining the association between e-cigarettes and cancer risk, but we excluded qualitative studies. We summarized findings on three types of e-cigarette exposure: acute, short- to medium-term, and long-term. Additionally, we assessed whether the health effects differ between subgroup populations based on various sociodemographic factors, for which we also screened the previously included studies in the KCL review. Different risk-of-bias tools were used to assess the quality of the included human studies.

**RESULTS:**

We included 39 studies in the main analysis and 12 in the subgroup analysis. Of these, 2 were longitudinal observational studies, 9 were cross-sectional studies, 1 case report and 27 were cell/*in vitro* and animal studies. All human studies were conducted in adults, and about half of them had a low risk of bias. No significant incident or prevalent risk of lung cancer or other types of cancer was found in the never smoker current vapers population. However, there was substantial biomarker-based evidence of a significant association between e-cigarette exposure and oxidative stress, cellular apoptosis, DNA damage, genotoxicity, and tumor growth, particularly following acute exposure. We did not find any age or sex-based differences in cancer risk, and findings on race and education-based differences were insufficient.

**CONCLUSIONS:**

There is substantial evidence that e-cigarette exposure is associated with biomarkers reflective of cancer disease risk. However, the overall evidence on cancer risk is still limited and should be further investigated by future research, particularly rigorously designed clinical trials and population-based research.

## INTRODUCTION

Electronic cigarettes (e-cigarettes) are devices that facilitate vaping aerosols from e-liquid that consists of nicotine and other chemicals. Despite coming onto the North American market recently, the use of e-cigarettes has risen at an alarmingly rate, particularly among young adults^1^. For example, the Canadian Tobacco and Nicotine Survey in 2021 reported that 5.3 million Canadians aged ≥15 years had ever used e-cigarettes and 1.6 million vaped in the past 30 days. The prevalence of ever vaping was highest in young adults aged 20–24 years, at 43%, while youth aged 15–19 years had a prevalence of 35%^2^. Despite their popularity, the short- and long-term health effects of e-cigarettes remain unclear. While e-cigarettes are marketed as a safer, healthier alternative to smoking combustible cigarettes, recent research has highlighted the wide-ranging, harmful health effects of vaping^3-5^. While it is generally understood that traditional smoking can result in cancer due to the carcinogens contained in tobacco cigarette smoke, whether e-cigarettes can also contribute to the development of cancer remains unknown. Although nicotine itself is not considered a carcinogen, e-cigarette aerosol does contain chemicals linked to cancer, such as formaldehyde and acrolein, heavy metals, and volatile organic compounds^3-5^. Therefore, theoretically, e-cigarette aerosol can potentially result in mutations in human DNA^4^. Additionally, biological pathways through which e-cigarette aerosols can contribute to cancer have been revealed in many studies. For example, compounds present in e-cigarette aerosols can lead to the generation of reactive oxygen species (ROS) and the creation of reactive intermediates that can bind to and damage DNA. Formaldehyde specifically can result in the binding of reactive molecules to DNA, which is a key part of chemical carcinogenesis. Of particular concern is DNA damage due to formaldehyde in the upper airways, as this damage could potentially contribute to the risk of nasopharyngeal and lung cancers specifically^3,4^. Nevertheless, the National Academies of Science, Engineering, and Medicine (NASEM) review^4^ concluded that it is yet to be determined whether the level of exposure to these chemicals is high enough to induce a carcinogenetic process. Additionally, although the recently published Kings College London (KCL)^5^ review did not find conclusive evidence linking e-cigarette use to cancer, they found biomarker-based evidence that the risk of cancer from vaping e-cigarettes might be greater than non-users but lower than smokers. As the research on the health effects of vaping is still emerging, findings are constantly being updated, and given the new findings, there is a need for conducting an updated review. Hence, we conducted this systematic review to amalgamate the available evidence to understand better the implications of e-cigarette exposure on the risk of developing cancer and update the findings of the KCL review^5^.

## METHODS

This review was conducted as part of the larger project ‘Vaping and Electronic Cigarette Toxicity Overview and Recommendations (VECTOR)’ to evaluate various health risks of vaping e-cigarettes in different populations. The protocol of this project was registered on the PROSPERO (registration no. CRD42023385632) (https://www.crd.york.ac.uk/prospero/display_record.php?RecordID=385632).

We followed the Preferred Reporting Items for Systematic Reviews and Meta-Analyses (PRISMA) guidelines, adhering to the 4-phase flow diagram and the 27-item checklist for this study (see PRISMA reporting checklist)^6^. We formulated three research questions for this review:

1. ‘Does e-cigarette or vaping product use (active and secondhand) increase the risk of cancer?’;
2. ‘How does the risk of cancer differ between people who vape but have never smoked, people who quit smoking but have continued vaping, and people who are both smoking and vaping (dual use)?’; and
3. ‘Does the cancer risk differ by age group, sex, gender, sexual orientation, race, ethnicity, indigenous identity, pregnant/postpartum, education level, individual or household income, employment status, and occupation?’.

We also aimed to compare our findings with the KCL review^5^, identify existing research gaps, and provide directions for future research.

### Information sources and search strategy

We searched the following databases: CINAHL, Embase, MEDLINE, PsycINFO, PubMed, and Cochrane Library. The literature search was conducted under the VECTOR project, where we used a broader search strategy covering articles for other health effects of vaping. We used a combination of various medical subject headings (MeSH) terms and keywords, and the detailed search strategies for different databases are presented in Supplementary file Material 1. As the recently published Kings College London (KCL) review^5^ included studies on cancer effects until June 2021, we limited our search for studies published between July 2021 and December 2023. However, the KCL review^5^ did not analyze the subgroups as stated in research question 3. So, we reviewed the 427 original studies included in the KCL review to assess whether they meet the eligibility criteria for subgroup analysis on cancer risk. All search results were imported to the Covidence workflow platform where duplicate articles were removed automatically.

### Eligibility and study selection process

We included studies that assessed the risk of cancer from exposure to e-cigarette aerosols or, e-liquids, or nicotine-containing vaping products (first and secondhand exposure) in either humans, cells (i.e. *in vitro* studies conducted on human or animal cells), or animals. Only peer-reviewed literature (published or in press) published in the English or French language was included. Hence, we excluded non-peer-reviewed literature (e.g. posters, conference abstracts, PhD theses). Other exclusion criteria were studies evaluating other health effects of vaping, qualitative studies, and literature reviews, studies evaluating the effects of cannabis vapor exposure or exposure to heated tobacco products or other tobacco products. No studies were excluded based on the sample size, participant socioeconomic status, country of origin, or presence or absence of a comparison group.

Studies were included in the subgroup analysis if they assessed the risk of cancer in different age groups, sex, gender, sexual orientation, race, ethnicity, indigenous identity, pregnant/postpartum population, education level, individual or household income, occupation, or employment status in the sample. For this purpose, we screened studies retrieved from our search and those that were finally included in the KCL review^5^ (n=427). We also included single-sex studies, such as animal studies conducted only in male or female sex or any case report conducted on a single individual in the sex-based subgroup analysis.

At least two reviewers independently screened each title and abstract, followed by full texts of the remaining articles in accordance with the inclusion and exclusion criteria. One reviewer resolved disagreements upon discussion with or guidance from other reviewers.

### Data collection process, data items, and effect measures, data synthesis

A custom-made data extraction form was developed, which included the following data items: general characteristics of the included studies (author and year, country, funding source, conflicts of interest, study design), population characteristics (sample number, demographics; sample’s status of e-cigarette use, cigarette use or dual use), e-cigarette exposure (type of exposure and duration of exposure), intervention/grouping’s characteristics (total number of participants in each intervention or comparison group, details of the exposure received), health condition/outcome assessed, reversibility of health effects, study findings, subgroup characteristics (type of subgroup, sample number), subgroup findings, risk of bias or critical appraisal scores for each study. By keeping consistent with the KCL review^5^, we assessed the health effects of 3 different types of e-cigarette exposure: acute (one-off exposure to 7 days), short- to medium-term (8 days to 12 months), and long-term exposure (more than 12 months). As cross-sectional studies measure outcomes at a single point, we did not mention any exposure type for these studies. For consistency and easy comparison, we categorized the comparison groups according to their exposure type and frequency of exposure. For example, we categorized e-cigarette users as non-smoker current vapers (individuals who used only e-cigarettes but not cigarettes in the past 30 days), never smoker current vapers (individuals who vaped in the past 30 days but never smoked or smoked <100 cigarettes in their lifetime), former smoker current vapers (previous smokers who used e-cigarettes in the past 30 days), and dual users (using both cigarettes and e-cigarettes in the past 30 days).

Similarly, we made other categories like non-vaper current smokers, never vaper current smokers, former vaper current smokers, non-users (used no cigarettes or e-cigarettes in the past 30 days), and never users (used <100 cigarettes and never used e-cigarettes in their lifetime). We considered diagnosed cancer, deoxyribonucleic acid (DNA) damage, mutagenesis, and carcinogenesis as irreversible health conditions. However, if the evidence of reversibility was not provided by the study that assessed molecular or biomarker-based changes, we considered it ‘not measured’. We measured the effects as risk ratio (RR), odds ratio (OR), effect sizes, incidence, prevalence, standard errors, or confidence intervals as reported in the included studies. We considered a p<0.05 or 95% confidence interval (CI) not containing the null value as a significant association. Depending on this significant association, we determined the direction of effect for risk of cancer as ‘no risk’, ‘increased risk’, and ‘inconsistent’.

A subset of the final included studies was tested first among two reviewers using the sample data extraction form. Once a good level of agreement had been achieved, the final data extraction form was used to extract data on the rest of the studies. While one reviewer extracted data, another reviewer checked for the accuracy of the extracted data for all finally included studies. Due to inconsistent comparison groups, different outcome measurement methods, and a low number of human studies, we could not perform any meta-analysis in this review. However, we followed a combination of synthesis without meta-analysis (SWiM)^7^ and narrative data synthesis approach^8^ to present our findings. In addition to summarizing the general characteristics of the studies, we demonstrated the distribution of the studies by study designs, risk of bias, risk of cancer, type of exposure, and subgroup findings by harvest plots^7^.

### Quality assessment

Quality assessment of individual studies was completed independently by two reviewers, and any disagreements were resolved by discussion between the reviewers. We used different risk-of-bias assessment tools depending on the study designs. The Risk of Bias in Non-randomized Studies- of Exposure (ROBINS-E) tool was used^9^ for longitudinal observational studies. For cross-sectional studies, we used the BIOCROSS risk of bias tool^10^ when it involved assessment of the health effects by biomarkers. Otherwise, we used the Joanna Briggs Institute (JBI) critical appraisal tool for analytical cross-sectional studies^11^. For case reports, we also used the JBI critical appraisal tools^11^. Consistent with the KCL review^5^, we did not assess the quality of the cell/*in vitro* and animal studies. As the KCL review^5^ has already conducted its own quality assessment for its studies, we did not conduct any separate quality assessment for the studies retrieved from the KCL review for subgroup analysis.

The quality assessment in the ROBINS-E tool is presented as ‘low’, ‘some concerns’, ‘high’, and sometimes ‘very high’ risk of bias^9^, The BIOCROSS tools include ten items, and the score could be between 0 to 210 for each item. By following approaches applied in previous research^12^, we considered ‘low’ risk of bias if the total score was 13–20, ‘moderate’ risk of bias if the score was 7–12, and ‘high’ risk of bias if the score was ≤6. The JBI critical appraisal tools for cross-sectional and case reports contain 8-item checklists, while the JBI tool for case series includes a 10-item checklist^11^. For each item appraised, we assigned a score of 1 if the criterion was met and 0 if the criterion was not met or was unclear. We followed approaches applied in previous research^13^ and considered a study having ‘low’ risk of bias if the total score was ≥70%, ‘moderate’ risk of bias if the score was 50–70%, and ‘high’ risk of bias is the score was <50%.

### Validation of the KCL review

As we included studies from the KCL review^5^ for subgroup analysis, we aimed to replicate their review’s study selection and data extraction process. We repeated their search strategy in MEDLINE, using their search period between August 2017 and July 2021. We screened the first 1000 search results based on our eligibility criteria and compared the included studies with those selected by the KCL review^5^. To validate the data extraction process, we randomly selected 40 studies from the 427 original studies included in the KCL review^5^ using a randomization tool. Next, we conducted the full-data extraction on these 40 studies to assess whether our findings match their results.

## RESULTS

### Study selection

**Figure 1:**
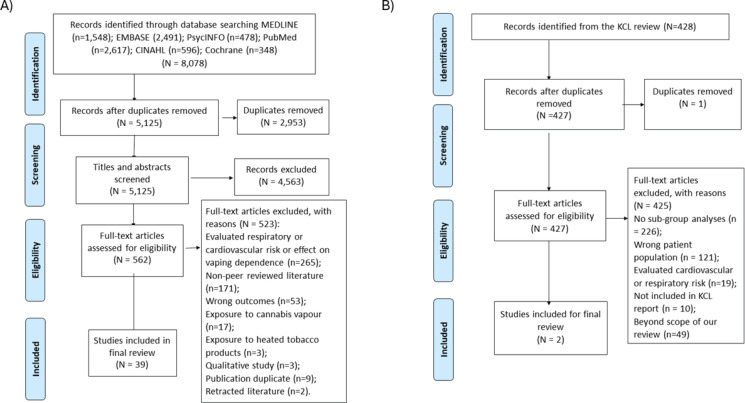
PRISMA flow diagram showing study selection process for (A) main analysis and (B) from the KCL review for the subgroup analysis

As part of the VECTOR systematic review, we retrieved 8078 articles from the databases. After removing 2953 duplicates, we screened titles and abstracts of 5125 articles, of which 562 were selected for full-text screening. After removing 523 articles for various reasons (Figure 1), 39 studies^14-52^ were selected for inclusion in this review. We note that we excluded studies that investigated other outcomes of the VECTOR systematic review during the full-text screening rather than in the title and abstract screening. Additionally, from the KCL review^5^, we screened full texts of 427 of their included studies for sub-group analysis. After removing 425 studies for various reasons (Figure 1), 2 studies^53,54^ were finally selected for inclusion in this study.

### Study characteristics

More than half of the 39 studies^14-52^ included in the main analysis were conducted in the United States (n=21; 54%) (Table 1). Twenty-three studies^18,22,25-29,31,33,35-37,39,40,42-48,50,52^ (59%) evaluated the effects of acute exposure, nine studies^15,17,26,29,33,34,41,49,51^ (23%) short-to-medium term exposure, and one study^20^ (3%) examined effects of long-term exposure. Only two studies (5%) were longitudinal observational studies^17,20^, while 9 (23%) were cross-sectional studies^14,16,19,21,23,24,30,32,38^, 1 (3%) was a case report^15^, and 27 (69%) were cell/*in vitro* or animal studies^18,22,25-29,31,33-37,39-52^. Acute and short-to-medium-term exposures were mainly studied by cell/*in vitro* and animal studies^18,22,25-29,31,33-37,39-52^, while a single longitudinal study^20^ examined long-term exposure. All 12 human studies^14-17,19-21,23,24,30,32,38^ were conducted in people aged ≥18 years. Eight out of these 12 studies had a sample size of 100–10000. We categorized the health outcomes under two main categories: lung cancer and any cancer, of which lung cancer was the most commonly investigated outcome (n=27; 69%). A total of 12 studies^15–17,21,24,26,29,49,51-54^ were included in the subgroup analysis, of which two studies^53,54^ were retrieved from the KCL review^5^. Four of these 12 studies assessed age-based differences^16,17,21,24^; all studies were included in sex-based analysis^14-16,20,23,25,28,48,50-55^, two studies were in race/ethnicity^21,24^, and one study in education-based subgroup analysis^24^ (Table 1). Characteristics of each study are presented in the Supplementary file Materials 2 and 3.

**Table 1:** Summary statistics of included studies examining cancerous effects of vaping e-cigarettes (N=39)

*Characteristics*	*Number* *of studies* *(%)*
**Outcomes/health condition(s)**	
Lung cancer	27 (69)
Any type of cancer	14 (36)
**Country**	
USA	21 (54)
Canada	2 (5)
European countries	8 (21)
Other	8 (21)
**Type of exposure**	
Acute	23 (59)
Short to medium	9 (23)
Long	1 (3)
**Study design**	
Longitudinal observational	2 (5)
Cross-sectional	9 (23)
Case report	1 (3)
Animal studies	8 (21)
*In vitro*/cell studies	21 (54)
**Number of participants** (human studies only, N=12)	
<100	3 (25)
100–1000	4 (33)
1001–10000	4 (33)
>10000	1 (8)
**Age of the participants** (human studies only, N=12)	
<18 years	0 (0)
≥18 years	12 (100)
**Risk of bias** (human studies only, N=12)	
Low	6 (50)
Moderate/some concerns	5 (42)
High/very high/serious/critical	1 (8)
**Association with tobacco companies**	
Yes	2 (5)
No	34 (87)
Not specified	3 (8)
**Subgroup analysis** (N=12)*	
Age	4 (33)
Sex	12 (100)
Ethnicity/race	2 (17)
Education level	1 (8)

* Subgroup analysis included studies from both this review (n=10) and the King’s College London review (n=2).

### Quality assessment

Of the 12 human studies, 6 (50%) had a low risk of bias^16,20,24,30,32,38^, 5 (41.7%) had some concerns or moderate risk^14,15,19,21,23^, and 1 (8.3%) had a very high risk of bias^17^. The very high risk of bias study was a longitudinal cohort study^17^, while the moderate risk of bias studies were cross-sectional studies and case reports. Only two of the included studies were funded by or had associations with tobacco companies^41,45^, while three studies^23,24,37^ did not specify whether they had such association (see Table 1 and Supplementary file Material 4). Our findings on the validation of the KCL review^5^ found minor discrepancies, which did not impact the validity of the study selection process and interpretation of the studies in the data extraction. The detailed findings on the validation process are presented in the Supplementary file Material 5.

### Lung cancer

**Figure 2:**
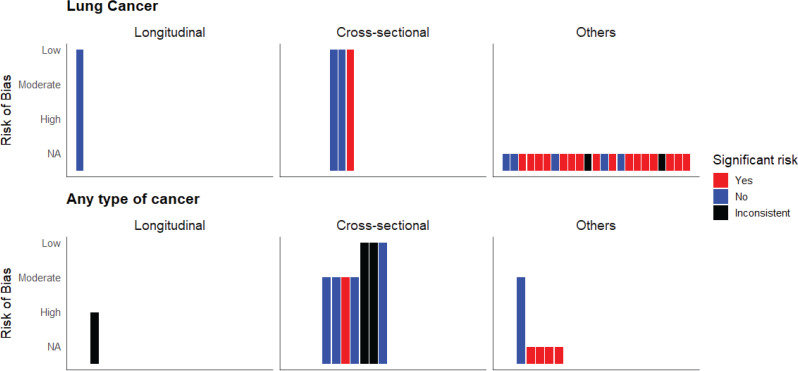
Harvest plot showing distribution of studies by risk of cancer, study designs, and risk of bias (individual bar in the plot represents a single study; ‘others’ study design includes case report, cell/*in vitro*, animal studies; risk of bias ‘NA’ indicates ‘not applicable’)

**Figure 3:**
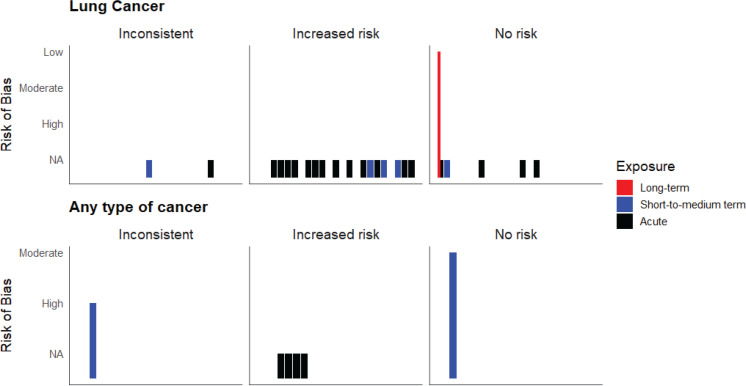
Harvest plot showing distribution of studies by risk of cancer, exposure type, and risk of bias (individual bar in the plot represents a single study; risk of bias ‘NA’ indicates ‘not applicable’)

We found one longitudinal observational study^20^, three cross-sectional studies^30,32,38^, eight cell/*in vitro* studies^22,25,33-37,39^, and three animal studies^28,29,33^ that addressed the risk of lung cancer associated with e-cigarette exposure. Overall, the majority of the longitudinal and cross-sectional studies did not find any significant risk of cancer. In contrast, studies with other study designs mostly suggested a higher risk of lung cancer following e-cigarette exposure (Figure 2). Focusing on the type of exposure, most of the studies that reported increased risk of lung cancer mainly assessed the effect of acute exposure to e-cigarettes (Figure 3). The longitudinal study is the only study that looked into long-term exposure and assessed the risk of lung cancer in a cohort of 119593 participants. The study reported that people with lung cancer were more likely to be former or current e-cigarette users (including dual users) compared to those without any lung cancer. However, they did not find any significant association between e-cigarette use and incident risk of lung cancer^20^. Of the cross-sectional studies, one study measured salivary DNA methylation score for lung cancer and did not find any apparent risk among never smoker current vapers^30^. Wharram et al.^38^ reported no significant difference in the likelihood of ever vaping among localized and advanced-stage lung cancers. Only one cross-sectional study reported that non-smoker current vapers had faster lung aging than never smokers, and the effect was similar to that seen among non-vaper current smokers, suggesting that non-smoker current vapers had an increased risk of age-related lung disease, including cancer^32^.

Among the cell/*in vitro* and animal studies, significant reduction in cell viability and increased apoptosis (p<0.05) were observed following acute exposure to nicotine e-cigarette exposure in 3 studies^22,48,52^, and non-nicotine e-cigarettes in one study^25^. One study only found a significant effect in diabetic mice compared to non-diabetic mice^52^. Two cell/*in vitro* studies did not find any significant effect on cell viability and apoptosis^37,39^, while another study found significantly decreased (p<0.05) cell proliferation and metabolic activity following acute exposure, indicating lower susceptibility to carcinogenesis^43^. Significant increases (p<0.05) in oxidative stress, such as increased reactive oxygen species production (ROS) or oxidative biomarkers expression, were found following acute exposure to nicotine e-cigarettes in 8 studies^22,29,44-47,50,52^, and following short-to-medium term exposure to nicotine e-cigarettes in 4 studies^29,34,49,51^. One of these studies found a significant effect only following e-cigarette exposure through a high-powered device^29^ and another following e-cigarette exposure only in diabetic mice^52^. Three studies also found significant oxidative stress (p<0.05) following acute exposure to non-nicotine e-cigarettes^46-48^. DNA damage or strand breaks (p<0.05) were observed following acute exposure to non-nicotine e-cigarettes in one study^25^ and nicotine e-cigarette exposure with high puff fraction in another study^22^. An animal study found a significantly increased (p<0.05) level of DNA damage following short-to-medium-term exposure to e-cigarettes delivered by high-powered devices^29^. In contrast, no significant effect on DNA damage, DNA methylation, or histone modulation was reported by two cell/*in vitro* studies following acute exposure^34,39^. Significant increases in genotoxicity or expression of proteins involved in mutagenesis were found following acute exposure to nicotine e-cigarette in 6 studies^28,34-37,42^, following short-to-medium term exposure in 1 study^33^, and following non-nicotine e-cigarette exposure in 1 study^25^. Two of these studies also reported a significant increase in cell transformation^33,34^. However, three studies did not find any significant evidence of genotoxicity following nicotine e-cigarette exposure^33,40,41^. One study detected significant levels (p<0.05) of toxic metals such as lead, copper and chromium in the e-cigarette aerosol^22^.

*Subgroup findings*

Only four single-sex-based animal studies^29,49,51,52^ were found analyzing oxidative stress in the lung, of which three studies were conducted in male mice^29,49,51^ and one study in female mice^52^. All studies reported a significant increase (p<0.05) in oxidative stress following nicotine e-cigarette exposure, increasing susceptibility to lung cancer.

### Any type of cancer

We found one longitudinal observational study^17^, seven cross-sectional studies^14,16,19,21,23,24,38^, one case report^15^, and four cell/*in vitro* and animal studies^18,26,27,31^ examined the risk of any cancer. The longitudinal study had a high risk of bias^17^, and three of the six cross-sectional studies^14,19,23^ and the case report^15^ had a moderate risk of bias. Overall, out of the eight longitudinal and cross-sectional studies, only one found an increased cancer risk. In contrast, the majority of the studies with other study designs reported a higher risk of cancer following e-cigarette exposure (Figure 2). None of the studies looked into long-term exposure. All studies that reported an increased risk of cancer assessed acute exposure effects to e-cigarettes (Figure 3). The longitudinal study assessed levels of carcinogens in the urine of 126 participants following short-to-medium term exposure and reported that levels of all carcinogens were significantly lower (p<0.001) in non-smoker current vapers compared to current smokers. However, they also reported significantly higher levels (p<0.001) of urinary acrolein metabolites in non-smoker current vapers compared to never users^17^. Among the cross-sectional studies, three studies did not find any significant association of ever vaping with a prevalence of non-melanoma skin cancer^19^, bladder cancer^16^, and any cancer^14^, respectively. One study reported significantly higher odds of being ever vapers among females diagnosed with metastatic breast cancer compared to those with localized breast cancer. However, this effect was not seen in the case of colorectal and prostate cancer^38^. Another study found that among current vaper cancer survivors, being former smokers or dual users was more likely than being never smokers, indicating a possible association with smoking rather than vaping^24^.

One study assessed salivary biomarkers (i.e. IL-1β and TGF-β). It concluded that non-smoker current vapers have significantly higher levels (p<0.001) of inflammatory and cancer risk biomarkers than non-vaper never smokers. In contrast, the level was significantly lower (p<0.001) than current smokers^23^. Similarly, another study looked into DNA damage markers in buccal cells and found significantly lower levels (p<0.05) in non-smoker current vapers compared to non-vaper current smokers^21^. Although we found one case report of Thoracic NUT (nuclear protein in testis gene) midline carcinoma in a male aged 33 years, the person had a 20-pack-year history of smoking and a recent history of vaping e-cigarettes. So, the association between e-cigarette use and diagnosis of this cancer is very weak^15^.

Among the four cell/*in vitro* and animal studies^18,26,27,31^, all showed that acute exposure to e-cigarettes promoted the growth of different cancers such as bladder cancer^27^, oral squamous cell carcinoma^18,31^, and brain tumor^26^. One also showed significantly accelerated growth (p<0.05) in brain tumors following short-to-medium-term e-liquid exposure^26^. Direct acute e-liquid exposure to normal oral epithelium cell lines showed significant cell viability^18^. Additionally, significantly increased oxidative stress, DNA damage, inflammatory markers of cell invasion, cell transformation, and apoptosis following acute exposure in cancer cells were observed in 3 studies^18,27,31^.

*Subgroup findings*

We found one longitudinal observational study^17^ and three cross-sectional studies^16,21,24^ examining age-based differences in any cancer risk following e-cigarette exposure. While the longitudinal study^17^ and two cross-sectional studies^16,21^ did not find any age-based differences, one cross-sectional study reported that younger cancer survivors had significantly higher odds of being current vapers compared to older cancer survivors^24^.

**Figure 4:**
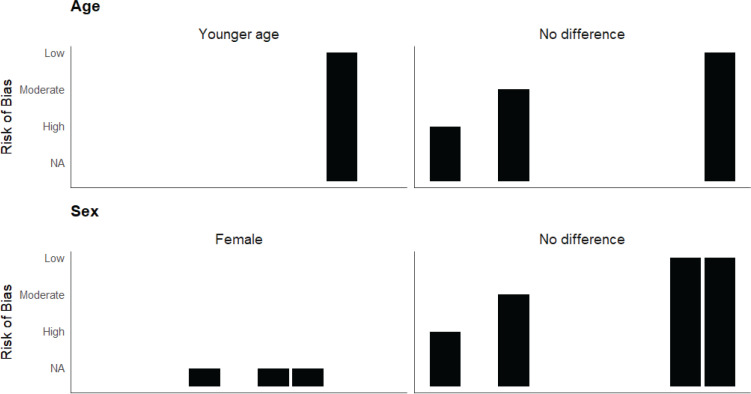
Harvest plot of studies with risk of any type of cancer demonstrates higher number of studies reporting no significant difference between age and sex subgroups (individual bar in the plot represents a single study; risk of bias ‘NA’ indicates ‘not applicable’)

Among the nine studies included in sex-based subgroup analysis, 1 was a longitudinal observational study^17^, 3 were cross-sectional studies^16,21,24^, 1 was a single-sex-based case report^15^, and 4 were single-sex based animal studies^26,29,53,54^. Of these, the longitudinal and cross-sectional studies did not find any sex-based differences in risk of any cancer^16,17,21,24^. The case report was on a male patient who was diagnosed with Thoracic NUT midline carcinoma^15^. Three of the four single-sex animal studies reported a significantly higher risk of cancer growth (i.e. breast cancer, brain tumor) following exposure to e-cigarettes in female mice^26,53,54^. Another study conducted in male mice found that e-cigarette exposure from a high-powered device significantly increased oxidative DNA damage in the lung and liver^29^. Depending on the direction of effect reported in the majority of the studies, we concluded that there were no significant age or sex-based differences in the risk of any cancer following e-cigarette exposure (Figure 4).

Of the two cross-sectional studies examining race-based differences, one found no significant findings^21^. In contrast, another study reported that Black and Asian cancer survivors had significantly lower odds (p<0.05) of current vaping compared to White cancer survivors^24^. They also reported that cancer survivors with less education had significantly higher odds (p<0.05) of being current vapers as opposed to cancer survivors with more education^24^.

## DISCUSSION

This systematic review reports important updates on the potential cancer outcomes related to vaping e-cigarettes. Overall, in line with what the KCL review^5^ found, recent studies do not definitively indicate that vaping e-cigarettes is linked to increased cancer risk. None of the included human studies reported any significant incident risk or prevalent risk of lung cancer or other type of cancer in never smokers current vapers. Nevertheless, a significant number of biomarker-based studies, cell/*in vitro* and animal studies did indicate that e-cigarette exposure can result in oxidative stress, cellular apoptosis, DNA damage, genotoxicity, and tumor growth (Figure 2, and Supplementary file Materials 2 and 3), all of which can potentially increase risk of developing lung cancer or progression of any cancer. However, most of these studies focused on acute exposure findings, leaving the cancer risk from short-to-medium- or long-term e-cigarette exposure unknown (Figure 3). Findings from the subgroup analysis were sometimes mixed, but overall, we did not find any notable age or sex-based differences in the risk of cancer following e-cigarette exposure (Figure 4).

### Limitations

A major limitation to concluding the current body of research on the cancer-related effects of e-cigarette use is the fact that, compared to other health effects, there is a lack of published studies on this topic. Another major limitation is that, due to the novelty of e-cigarettes and their popularity mainly among young adults, there is a lack of long-term population-level data on cancer risk. We found only one study examining long-term exposure using prospective data of only 2 years^20^, which is not enough for developing cancer. While cancer usually has a long latency period (i.e. 20 years for lung cancer^55^) and hence, long-term follow-up of e-cigarette users should be initiated, we should also consider other methods, such as a presumptive period method to measure the incident risk of cancer following vaping cessation to understand effects of long-term exposure^55^. Additionally, biomarker-based evidence of cancer risk can be easily investigated through rigorously designed randomized controlled trials to assess short-to-medium-term exposure effects. Unfortunately, the biomarker-based evidence in our review was not investigated through any human clinical trial. Translating the biomarker-based evidence in clinical settings would allow us to understand better and predict the long-term risk of cancer from e-cigarette exposure^56^, which will, in turn, provide policymakers with meaningful results. Other limitations include methodological inconsistencies in the studies included in this review. For instance, some studies defined one of their population groups as ‘ever vapers’^14,16^, which is misleading because this can include people who could have used e-cigarettes currently or sporadically anytime in their lives. It might be inappropriate and misinformative to the audience to indicate the risk of cardiovascular effects in current vapers, whereas this effect might be from smoking cigarettes rather than e-cigarettes. Although we included studies published since the KCL review^5^ and our findings did not differ, there are some methodological differences between these two reviews. First, unlike the KCL review^5^, we introduced different sociodemographic factor-based subgroup analyses in our study.

In addition to biomarker-based evidence, we also included self-reported data, like the incidence or prevalence of cancer outcomes and case reports, in this review. We thought this information was important to understand population trends and included these studies. Moreover, the KCL review mostly defined non-use as not smoking or vaping in the past six months and daily or almost daily use as current use; we defined our comparison groups differently to reflect the definition of the current use (use in the past 30 days) that were used in the majority of the studies. Hence, these differences should be considered when comparing our findings with those of the KCL review^5^. We did not conduct any meta-analysis in our review due to the significant heterogeneity between the studies and the lack of human-based evidence. However, we conducted synthesis without meta-analysis (SWiM) and presented several harvest plots to demonstrate our findings (Figures 2–4). We also identified relevant research gaps, which will guide future researchers to conduct rigorously designed human studies and meta-analysis examining risk of cancer from e-cigarette use.

## CONCLUSIONS

While research to date does not provide evidence of any significant risk of lung cancer or other types of cancer from e-cigarette use, we found substantial biomarker-based evidence. The body of research is severely limited by the number of studies, study designs, heterogeneity of the studies, and quality of evidence. Hence, there is a need for updating the evidence and investigating further risk of cancer from e-cigarette exposure by conducting high-quality prospective longitudinal studies.

## Supplementary Material



## Data Availability

The data supporting this research can be found in the Supplementary file.
