# Supplementary material for: Evidence update on the cancer risk of vaping e-cigarettes: A systematic review
Source: Tob Induc Dis. 2025 Jan 28;23:10.18332/tid/192934. doi: 10.18332/tid/192934 (PMC11773639; doi:10.18332/tid/192934)
Supplement: Supplementary file 1 [file TID-23-06-s1.pdf]

Supplemental Materials

Evidence update on the cancer risk of vaping e-cigarettes: A systematic review.

Supplemental Material 1. Data base search strategy

Supplemental Material 2. Summary of studies assessing risk of cancer from exposure to e-cigarettes (n=39)

Supplemental Material 1. Summary of studies retrieved from the KCL review for subgroup analysis on risk of cancer from exposure to e-cigarettes (n=2)

Supplemental Material 4. Quality assessment findings

Supplemental Material 5. Validation of the KCL review

Supplemental Material 1. Data base search strategy

Date	Serial	Searches	Results
**MEDLINE**			
Jan 31, 2023	1.	1. exp Electronic Nicotine Delivery Systems/<br>2. exp Vaping/<br>3. e-cig*.tw,kw.<br>4. electronic cig*.tw,kw.<br>5. (ENDS and nicotine).tw,kw.<br>6. electronic nicotine delivery system*.tw,kw.<br>7. vaping.tw,kw.<br>8. vape*.tw,kw.<br>9. (nicotine and (vapor* or vapouris*)).tw,kw.<br>10. 1 or 2 or 3 or 4 or 5 or 6 or 7 or 8 or 9<br>11. exp Lung/<br>12. ((respiratory or lung) adj2 effect*).tw,kw.<br>13. exp Cardiovascular Diseases/<br>14. ((heart or cardiac or cardiovascular or vascular) adj2 effect*).tw,kw.<br>15. exp Neoplasms/<br>16. (neoplas* or cancer* or carcinoma* or malignan* or tumor*).tw,kw.<br>17. 11 or 12 or 13 or 14 or 15 or 16<br>18. 10 and 17<br>19. limit 18 to yr="2021 -Current"	359
Jan 31, 2023	2.	1. exp Electronic Nicotine Delivery Systems/<br>2. exp Vaping/<br>3. exp "Tobacco Use Disorder"/	891

		4. (e-cig* or (electronic adj2 cig*) or ENDS or vaping or vape*).tw,kw.<br>5. ((e-cig* or vaping or nicotine) adj2 (dependen* or addict*)).tw,kw.<br>6. 1 or 2 or 4<br>7. 3 or 5<br>8. 6 and 7<br>9. limit 8 to yr="2017 -Current"	
Jan 02, 2024	3.	Updated search 1<br>limit 18 to dt=20230201-20231231	152
Jan 02, 2024	4.	Updated search 2<br>limit 8 to dt=20230201-20231231	146
**EMBASE**			
Jan 31, 2023	1.2	1. exp Electronic Nicotine Delivery Systems/<br>2. exp Vaping/<br>3. e-cig*.tw,kw.<br>4. electronic cig*.tw,kw.<br>5. (ENDS and nicotine).tw,kw.<br>6. electronic nicotine delivery system*.tw,kw.<br>7. vaping.tw,kw.<br>8. vape*.tw,kw.<br>9. (nicotine and (vapor* or vapouris*)).tw,kw.<br>10. 1 or 2 or 3 or 4 or 5 or 6 or 7 or 8 or 9<br>11. exp Lung/<br>12. ((respiratory or lung) adj2 effect*).tw,kw.<br>13. exp Cardiovascular Diseases/<br>14. ((heart or cardiac or cardiovascular or vascular) adj2 effect*).tw,kw.<br>15. exp Neoplasms/	907

		16. (neoplas* or cancer* or carcinoma* or malignan* or tumor*).tw,kw.<br>17. 11 or 12 or 13 or 14 or 15 or 16<br>18. 10 and 17<br>19. limit 18 to yr="2021 -Current"	
Jan 31, 2023	2.	1. exp Electronic Nicotine Delivery Systems/<br>2. exp Vaping/<br>3. exp "Tobacco Use Disorder"/<br>4. (e-cig* or (electronic adj2 cig*) or ENDS or vaping or vape*).tw,kw.<br>5. ((e-cig* or vaping or nicotine) adj2 (dependen* or addict*)).tw,kw.<br>6. 1 or 2 or 4<br>7. 3 or 5<br>8. 6 and 7<br>9. limit 8 to yr="2017 -Current"	1362
Jan 02, 2024	3.	Updated search 1<br>limit 18 to dd=20230201-20231231	170
Jan 02, 2024	4.	Updated search 2<br>limit 8 to dd=20230201-20231231	52
**PsycINFO**			
Jan 31, 2023	1.	1. exp Electronic Cigarettes/<br>2. e-cig*.tw.<br>3. electronic cig*.tw.<br>4. (ENDS and nicotine).tw.<br>5. electronic nicotine delivery system*.tw.<br>6. vaping.tw.<br>7. vape*.tw.<br>8. (nicotine and (vapor* or vapouris*)).tw.	49

		9. 1 or 2 or 3 or 4 or 5 or 6 or 7 or 8<br>10 exp lung disorders/<br>11 ((respiratory or lung) adj2 effect*).tw.<br>12 exp Cardiovascular Disorders/<br>13 ((heart or cardiac or cardiovascular or vascular) adj2 effect*).tw.<br>14 exp Neoplasms/<br>15 (neoplas* or cancer* or carcinoma* or malignan* or tumor*).tw.<br>16 10 or 11 or 12 or 13 or 14 or 15<br>17 9 and 16<br>18 limit 17 to yr="2021 -Current"	
Jan 31, 2023	2,	1. exp Electronic Cigarettes/<br>2. "tobacco use disorder"/<br>3. (e-cig* or (electronic adj2 cig*) or ENDS or vaping or vape*).tw.<br>4. ((e-cig* or vaping or nicotine) adj2 (dependen* or addict*)).tw.<br>5. 1 or 3<br>6. 2 or 4<br>7. 5 and 6<br>8. limit 7 to yr="2017 -Current"	336
Jan 02, 2024	3.	Updated search 1<br>limit 17 to up =20230201-20231231	28
Jan 02, 2024	4.	Updated search 2<br>limit 7 to up =20230201-20231231	65
**CINAHL**			
Jan 31, 2023	1.	S1. (MH "Electronic Cigarettes")<br>S2. (MH "Vaping")<br>S3. TI (e-cig* or (electronic N2 cig*) or ENDS or vaping or vape*)<br>S4. S1 OR S2 OR S3<br>S5. (MH "Lung Diseases")	339

		S6. TI ((respiratory or lung) N2 effect*)<br>S7. (MH "Cardiovascular Diseases")<br>S8. TI ((heart or cardiac or cardiovascular or vascular) N2 effect*)<br>S9. (MH "Neoplasms")<br>S10. TI (neoplas* or cancer* or carcinoma* or malignan* or tumor*)<br>S11. S5 OR S6 OR S7 OR S8 OR S9 OR S10<br>S12. S4 AND S11<br>S13. S12 ((Limiters - Published Date: 20210701-20221231)	
Jan 31, 2023	2.	S1. (MH "Electronic Cigarettes")<br>S2. (MH "Vaping")<br>S3. TI (e-cig* or (electronic N2 cig*) or ENDS or vaping or vape*)<br>S4. S1 OR S2 OR S3<br>S5. TI (e-cig* or vaping or nicotine) N2 (dependen* or addict*)<br>S6. S4 AND S5<br>S7.S6 (Limiters - Published Date: 20170101-20221231)	73
Jan 02, 2024	3	Updated search 1<br>S14. S12 ((Limiters - Published Date: 20230201-20231231)	171
Jan 02, 2024	4.	Updated search 2<br>S8. S6 ((Limiters - Published Date: 20230201-20231231)	13
**PubMED**			
Jan 31, 2023	1.	1. "electronic nicotine delivery systems"[MeSH Terms] OR ("electronic"[All Fields] AND "nicotine"[All Fields] AND "delivery"[All Fields] AND "systems"[All Fields]) OR "electronic nicotine delivery systems"[All Fields] OR ("electronic"[All Fields] AND "cigarettes"[All Fields]) OR "electronic cigarettes"[All Fields]<br>2. "vaped"[All Fields] OR "vaping"[MeSH Terms] OR "vaping"[All Fields] OR "vapes"[All Fields]<br>3. #1 OR #2<br>4. "lung diseases"[MeSH Terms] OR ("lung"[All Fields] AND "diseases"[All Fields]) OR "lung diseases"[All Fields]<br>5. "cardiovascular diseases"[MeSH Terms] OR ("cardiovascular"[All Fields] AND "diseases"[All Fields]) OR "cardiovascular diseases"[All Fields]<br>6. "cancer s"[All Fields] OR "cancerated"[All Fields] OR "canceration"[All Fields] OR "cancerization"[All Fields] OR "cancerized"[All Fields] OR "cancerous"[All Fields] OR "neoplasms"[MeSH Terms] OR "neoplasms"[All Fields] OR "cancer"[All Fields] OR "cancers"[All Fields]<br>7. #4 OR #5 OR #6<br>8. #3 AND #7<br>9. #8 Filters: from 2021/7/1 - 2022/11/28	937
Jan 31, 2023	2.	1. "electronic nicotine delivery systems"[MeSH Terms] OR ("electronic"[All Fields] AND "nicotine"[All Fields] AND "delivery"[All Fields] AND "systems"[All Fields]) OR "electronic nicotine delivery systems"[All Fields] OR ("electronic"[All Fields] AND "cigarettes"[All Fields]) OR "electronic cigarettes"[All Fields]<br>2. "vaped"[All Fields] OR "vaping"[MeSH Terms] OR "vaping"[All Fields] OR "vapes"[All Fields]	998

		3. #1 OR #2<br>4. "tobacco use disorder"[MeSH Terms] OR ("tobacco"[All Fields] AND "disorder"[All Fields]) OR "tobacco use disorder"[All Fields] OR ("nicotine"[All Fields] AND "dependence"[All Fields]) OR "nicotine dependence"[All Fields]<br>5. #3 AND #4<br>6. #5 Filters: from 2017/1/1 - 2022/11/28	
Jan 02, 2024	3.	Updated search 1<br>9. #8 Filters: from 2023/02/01 - 2023/12/31	520
Jan 02, 2024	4.	Updated search 2<br>7. #5 Filters: from 2023/02/01 - 2023/12/31	162
**Cochrane**			
Jan 31, 2023	1.	#1 [mh "Electronic Nicotine Delivery Systems"]<br>#2 [mh vaping]<br>#3 (e-cig* or (electronic NEAR/2 cig*) or ENDS or vaping or vape*):ti,ab,kw<br>#4 [mh "lung diseases"]<br>#5 ((respiratory or lung) NEAR/2 effect*):ti,ab,kw<br>#6 [mh "Cardiovascular Diseases"]<br>#7 ((heart or cardiac or cardiovascular or vascular) NEAR/2 effect*):ti,ab,kw<br>#8 [mh Neoplasms]<br>#9 (neoplas* or cancer* or carcinoma* or malignan* or tumor*):ti,ab,kw<br>#10 #1 or #2 or #3<br>#11 #4 or #5 or #6 or #7 or #8 or #9<br>#12 #10 and #11 with Cochrane Library publication date Between Jul 2021 and Nov 2022	92
Jan 31, 2023	2.	#1 [mh "Electronic Nicotine Delivery Systems"]<br>#2 [mh vaping]<br>#3 (e-cig* or (electronic NEAR/2 cig*) or ENDS or vaping or vape*):ti,ab,kw<br>#4 [mh "Tobacco Use Disorder"]<br>#5 ((e-cig* or vaping or nicotine) NEAR/2 (dependen* or addict*)):ti,ab,kw<br>#6 #1 or #2 or #3<br>#7 #4 or #5<br>#8 #6 and #7 with Cochrane Library publication date Between Jan 2017 and Nov 2022	185
Jan 02, 2024	3.	Updated search 1<br>#12 #10 and #11 with Cochrane Library publication date Between Feb 2023 and Dec 2023	51
Jan 02, 2024	4.	Updated search 2<br>#8 #6 and #7 with Cochrane Library publication date Between Feb 2023 and Dec 20231	20

Supplemental Material 2. Summary of studies assessing risk of cancer from exposure to e-cigarettes (n=39).

Author and year; country	Funding source; conflict of interest	Type of exposure; exposure length	Total participant	Intervention/ grouping(s)	Health condition; reversibility of health condition	Study findings	Subgroup	Subgroup findings	Risk of bias/ critical appraisal
**Longitudinal observational studies**									
Goldberg Scott et al., 2023;[20] US	Independent funding organization; none	Long-term; Prospective: Median days 504-713 days, Retrospective: EHR data.	N=119,593; 18+ years age, 22% >70 years; 60% female; Heterosexual (n=109,222); NH White (70%)	ENDS never user (n=112396, 94%)<br><br>ENDS former user (n=5603, 5%)<br><br>ENDS current user (n=1594, 1%): included dual users, non-smoker current vapers	Lung cancer; irreversible	Those with a history of lung cancer were more likely former and current ENDS users compared to those with no history of lung cancer (OR = 2.47, CI: 1.64-3.72; OR = 2.64; CI:1.42-4.92 respectively).<br><br>NS association between incident cases of any cancer or lung cancer with ENDS use.<br><br>Interpretation: Vaping was associated with increased risk of prevalence of lung cancer, but not incidence of lung cancer or any cancer.	N/A	N/A	Low
Chen et al., 2023;[17] US	Independent funding organization; Some (no pro-tobacco associations)	Short-to-medium term (4-6 months)	N=126; 18-67 years age; male (n=45), female (n=81); all in good physical and mental health, no infections.	Non-smoker current vapers (n=30): exclusively used EC and no Cigs for at least 3 months and at least 4 days per week, had exhaled CO <6 ppm.<br><br>Never users (n=63): smoked less	Any type of cancer; not measured	Urinary acrolein (3-hydroxypropyl mercapturic acid, 3-HPMA), benzene (S-phenyl mercapturic acid, SPMA), acrylonitrile (cyanoethyl mercapturic acid, CEMA), and 3-hydroxy-1-methylpropyl mercapturic acid (HMPMA) levels:<br>were significantly lower in non-smoker current vapers compared to current smokers (p <0.001 always).<br>However, compared to never users, non-smoker current	Age:<br>Mean age of Non-smoker current vapers: 32.7 yrs.,<br>Never users: 33.0 yrs,<br>Current smokers: 46.8 yrs.	Age:<br>NS association with age was found.<br><br>Sex:<br>NS difference was found between males and females.	Very high

				than 100 Cigs in their lifetime and never used any other tobacco products or ECs, had exhaled CO <6 ppm.<br><br>Current smokers (n=33): smoked at least 5 Cigs per day for a minimum of 4 days per week for the past year, had exhaled CO >8 ppm.<br><br>Urine sample was collected monthly over a period of 4-6 months.		vapers had significantly higher 3-HMPA level (p=0.003), other wise, NS difference in other chemical were seen between them.<br><br>Interpretation: Short-to-medium term exposure to EC was associated with significantly increased levels of acrolein metabolite in urine of non-smoker current vapers compared to never users, however, this level was significantly lower than current smokers. Otherwise, non-smoker current vapers had NS levels of other carcinogens in urine similar to never users.	Sex: Non-smoker current vapers: male (n=12), female (n=18), Never users: male (n=21), female (n=42), Current smokers: male (n=16), female (n=17).		
**Cross-sectional studies**									
Guo et al., 2021;[21] US	Independent funding organization; none	N/A	N=95; age >18 years	Non vaper current smokers: n = 30, smoked at least five Cigs/day for at least 4 days/week for the past year and had a carbon	DNA damage-initiation of carcinogenic process; irreversible	Apurinic/apyrimidinic (AP) site formation in DNA in buccal cell DNA:<br>Significantly lower among non-smoker current vapers (p<0.05) compared to non vaper current smokers and non users.<br><br>Interpretation: DNA damage markers was significantly lower among non-smoker current	Age groups: ≤30 years, 30-50 years, >50 years<br><br>Sex and race groups: Not specified.	Age groups: NS difference in AP site formation between groups in ≤ 30 years and >50 years.	Moderate (11/20)

				monoxide (CO) >8 ppm.<br><br>Non-smoker current vapers: n = 30, exclusively used ECs for at least 3 months and at least 4 days/week and have a CO < 6 ppm<br><br>Non-users: n = 35, smoked no more than 100 Cigs in their lifetime and have a CO < 6 ppm.		vapers compared to non-vaper current smokers.		Sex and race:<br>NS differences were found among sex or race subgroups.	
Dugan et al., 2021;[19] US	None; none	N/A	N=3,454; mean age 67 years; 50% female; 98.5% White, 98.2% NH; all had non-melanoma skin cancer	Ever smokers: n= 1,630<br><br>Ever vapers: n= 247	Non-melanoma skin cancer; irreversible	Prevalence of nonmelanoma skin cancer:<br>NS risk was found among ever vapers.<br><br>Interpretation: Vaping does not seem to increase risk of non-melanoma skin cancer.	N/A	N/A	Moderate (5/8)
Kamal and Shams, 2022;[23] Egypt	Not specified; none	N/A	N=150; mean age 28-29 years.	NS/never smokers (n=50)<br><br>CS/current smokers (n=50): used Cig 14.7 ± 2.5 times/day,	Any type of cancer; not measured	Mean difference of salivary biomarkers between groups:<br>IL-1β:<br>CS - NS: 27.12906 (p<0.001)<br>CS - EC: 16.54890 (p<0.001)<br>EC - NS: 10.58016 (p<0.001)<br><br>TGF-β:<br>CS - NS: 131.22308 (p<0.001)	N/A	N/A	Moderate (11/20)

				each session 5.2 ± 0.8 mins; must have smoked for at least 1 year.<br><br>EC/non-smoker current vapers (n=50): used EC 10.1 ± 1.4 times/day, each session 7.8 ± 0.4 mins; must have vaped exclusively for at least 1 year.		CS - EC: 94.47308 (p<0.001)<br>EC - NS: 36.75000 (p<0.001)<br><br>Interpretation: EC users had significantly higher levels of inflammatory and cancer risk biomarkers than never smokers, but the level was significantly less than conventional cigarette users.			
Richmond et al. 4, 2021;[30] UK	Independent funding organization; none	N/A	N=350; 16-35 years age; 55.1% female, 44.9% male; 86% White.	Never smoker current vapers (n=116): used ECs at least weekly for the past 6 months and having smoked<100 times in their lifetime.<br><br>Never vaper current smokers (n=117): smoked at least weekly for the past 6 months and having used an EC <100 times	Lung cancer; irreversible	DNA methylation score:<br>DNA methylation at 7 CPGs sites associated with EC use were largely distinct from those associated with smoking.<br>DNA methylation score for EC use did not discriminate lung adenocarcinoma and squamous cell carcinoma from adjacent normal tissue.<br><br>Interpretation: There is apparently no raised risk of lung cancer from EC use among never smoker current vapers.	N/A	N/A	Low (15/20)

				in their life-time.<br><br>Never users (n=116): smoked and/or used an EC <100 times in lifetime.					
Wharram et al., 2023;[38] US	Independent funding organization; none	N/A	N = 4,443; mean age 60.9 years; 45.7% male, 54.3% female; all AA cancer survivors	Never smoker ever vapers (n=31): smoked<100 Cig in their lifetime and ever vaped EC.<br><br>Never users (n=2060): have used <100 Cigs in their lifetime and never vaped/smoked ECs in their lifetime.	Breast, prostate, colorectal, and lung cancer; irreversible	Breast cancer:<br>Those with metastatic breast cancer were significantly more likely to be ever vapers compared to those with localized breast cancer (aOR 2.37).<br><br>Colorectal, lung, and prostate cancer:<br>NS difference was seen in likelihood of ever vaping between localized and advanced stage of cancers.<br><br>Interpretation: Ever use of EC was significantly associated with metastatic stage of diagnosis of breast cancer. NS association was found between EC use and metastatic stage of lung, prostate, and colorectal cancer.	N/A	N/A	Low (8/8)
Song et al., 2023;[32] Germany	Independent funding; some (no pro-tobacco association)	N/A	N=69; ages 21-30 years; 46% female; 80% White, 20% Non-White	Non-smoker current vapers: EC (*n* = 14): Used EC daily for ≥1 year and had not smoked Cig for>6 months.	Lung cancer; irreversible	Lifespan and mortality were assessed by measuring DNA methylation (mAge) and its acceleration (mAA) in BALF: Non-smoker current vapers had significantly higher level of both mAge and mAA than never smokers (p=0.002).	N/A	N/A	Low (15/20)

				Non-vaper current smokers: NS, (*n* = 16): Used >10 Cigs per day for>6 months and had not used an EC for≥1 year.<br><br>Never smokers (n=39): smoked<100 Cig in their lifetime and had not used a EC for≥1 year.		NS difference between non-smoker current vapers and non-vaper current smokers.<br><br>Telomere shortening measurement in BALF:<br>Non-smoker current vapers had significantly lower level than never smokers (p=0.02).<br>NS difference between non-smoker current vapers and non-vaper current smokers.<br><br>Chronological aging measurement in BALF:<br>NS difference between groups.<br><br>Interpretation: Non-smoker current vapers had faster lung aging compared to never smokers, which is also similar to non-vaper current smokers, indicating high risk of age-related diseases like cancer.			
Catto et al., 2023;[16] Netherlands	Independent funding organization; no COI	N/A	N=2092; ≥18 years age; 77% male; 95% White; all participants were diagnosed with bladder cancer.	Ever vapers (n=165): used EC in their lifetime,<br><br>Never vapers (n=1668): never used EC in lifetime,<br><br>Ever smokers (n=1305): Currently smoking cigarettes/cigars/ pipes daily	Bladder Cancer; irreversible	Prevalence of ever vaping: Among participants with bladder cancer, prevalence of ever smoking (71%) was considerably higher than that of ever vaping (9%). Transition from smoking to vaping was more prevalent among current smokers than former smokers, most of which occurred after diagnosis.<br><br>Types of bladder cancer (muscle invasive bladder cancer vs non-muscle-invasive bladder cancer):	Age groups: Among ever and never users of EC (N=1,833)- 18-65 years: n=252, 65-74 years: n=707, 75-84 years: n=844, 86+ years:	Age: Among participants with bladder cancer, ever vaping was significantly (p<0.001) less prevalent than never vaping across all age groups.	Low (6/8)

				(n=194) + former smokers (n=1211) who quit smoking in last year,<br><br>Never smokers (n=566): Never smoked cigarettes/cigars/pipes.		NS difference was observed.<br><br>Interpretation: NS association was found between prevalence of bladder cancer and e-cigarette use.	n=289.<br><br>Sex: Among ever and never users of EC (N=1,833)- male: n=1610, female: n=482.	Sex: NS difference between male and female was observed.	
Austin-Datta et al., 2023;[14] US	Independent funding organization; no COI	N/A	N=7,253; ≥18 years age; 63.1% female; 48.4% Black/AA, 44.4% White, 92% non-Hispanic, 8% Hispanic.	Never vapers (n=6076): Never used EC in lifetime.<br><br>Ever vapers (n=1,177): Used EC in their lifetime.	Any type of cancer; irreversible	Prevalence of ever vaping: Odds of ever use was NS in those with cancer compared to those not having any cancer.<br><br>Interpretation: Ever vaping was not associated with higher prevalence of cancer.	N/A	N/A	Moderate (5/8)
Kim and Keegan, 2022;[24] US	Not Specified; None	N/A	N=2579; 18-≥75 years age; 57% female; 79% White. All individuals were cancer survivors.	Current vapers (n=74, 2.9%): Used EC everyday or some days.<br><br>Current smokers (n=302, 11.8%): Smoked Cig everyday or some days.	Any type of cancer; irreversible	Type of cancer: Among current vaper cancer survivors, breast cancer, cervical cancer, lung cancer and having more than one cancer had higher than average prevalence. However, the prevalence was lower than that seen among current smoker cancer survivors.<br><br>Among current vaper cancer survivors, being former smokers or dual users had significantly higher odds (aOR 14.82 and 58.39 respectively) than being never smokers.	Among current vapers cancer survivors,<br><br>Age groups: 18-34 yrs (0%), 35-49 yrs (6.7%), 50-64 yrs (2.1%), 65-74 yrs (2.8%), ≥75 yrs (1.8%)	Age: Younger cancer survivors (18-34 yrs, and 65-74 yrs) had significantly ($p<0.05$) higher odds of current vaping than older (≥75 yrs) cancer survivors.<br><br>Sex: NS	Low (7/8)

						Interpretation: The prevalence of current EC use was lower than current smoking among cancer survivors, and most of the current vapers were former smokers or dual users.	Sex: male (1.9%), female (3.7%).<br><br>Race/ethnicity: White (3.5%), Black/AA (0.2%), Hispanic (4%), Asian (0%), other race/ethnicity (0.6%)<br><br>Education: Less tha high school (6.2%), high school graduate (2.7%), some college (4%), college graduate or more (1.1%).	difference was seen between males and females.<br><br>Ethnicity: Black/AA and Asian had significantly lower odds ($p<0.05$) of current vaping than White cancer survivors.<br><br>Education: Cancer survivors with less education (education lower than college graduate or more) had significantly higher odds of current vaping compared to those who had college graduates or more.	

**Case report**									
Ballenberger et al., 2022;[15] US	None; none	Short to medium term; 12 months	N=1; 33 years male; had 20 pack year smoking history; recently started ECs	N/A	Thoracic NUT (nuclear protein in testes gene) midline carcinoma; irreversible	Diagnosis: Thoracic NUT-midline carcinoma with metastases.<br><br>Interpretation: Patient was an active smoker and recently started ECs, So, outcome is not most likely due to using EC. Moreover, NUT midline carcinoma is a rare condition.	Sex: male (n=1)	Same as findings of entire population	Moderate (4/8)
**Cell/*in vitro* studies**									
Rayner et al., 2022;[40] US	Independent funding organization(s); none	Acute; Single exposure	Primary Normal Human Bronchial Epithelial (NHBE) cell cultures	Cig smoke conditioned media: exposed to low, medium, high doses of Cig for 0, 4 or 24 hrs.<br><br>Nic-EC Aerosol conditioned media: exposed to low, medium, high doses of tobacco flavored 1.8% nicotine EC for 0, 4 or 24 hrs.<br><br>PG/VG: exposed to PG/VG (no nicotine) for 0, 4 or 24 hrs.	Lung genotoxicity; Not measured	Gene expression for oxidative stress, xenobiotic metabolism, SPINK1 general cancer pathways and mucociliary clearance:<br>No changes were observed with Nic-EC preparations containing up to 28 µg/mL nicotine. NS difference between Ni-EC and PG/VG control.<br><br>Interpretation: Acute exposure to nicotine EC had no apparent toxicogenomic effect in lung cells.	N/A	N/A	

				Cells taken from 4 donors, 3 were non-smokers and one had unknown smoking status.					
Rayner et al., 2022a;[41] US	Tobacco industry and university funding; many (association with vape industry)	Short to medium term; 10 days	Primary normal human bronchial epithelial (NHBE) cells.	Control group (cells from n=3 donors)<br><br>Nic-EC group (cells from n=3 donors): Exposed to EC aerosol with1.8% nicotine in Low and high concentrations for 1 hr./day for 10 days.<br><br>Cig Smoke group (cells from n=3 donors): Exposed to whole Cig smoke in Low and high concentrations for 1 hr./day for 10 days.	Lung genotoxicity; Not measured	Gene expression:<br>Nic-EC exposure at both low and high doses altered expression of 16 and 167 genes compared to control respectively. Only 9 genes were common between low and high doses of Nic-EC.<br>NS changes in cytokine/chemokine pathways, metabolism of xenobiotics by cytochrome P450, and chemical carcinogenesis were seen in Nic-EC exposure<br><br>Oxidative stress markers: NS changes seen on Nic-EC exposure.<br><br>Interpretation: Short-to-medium exposure to nicotine EC had no apparent toxicogenomic effect in lung cells.	N/A	N/A	
Noël et al., 2022;[44] The US	Independent funding organization(s); none	Acute; Single exposure	Human lung epithelium BEAS-2B cells	Nic-EC Strawberry flavoured: 18 mg/ml	Pulmonary oxidative stress; Not measured	Extracellular ROS level and NO level:<br>Strawberry flavoured Nic-EC exposure had increased	N/A	N/A	

				nicotine and 30/70 PG/VG.<br><br>Nic-EC Vanilla flavoured: 18 mg/ml nicotine and 30/70 PG/VG.<br><br>Control: exposed to air.		ROS/NO levels in cell media compared to control ($p<0.05$). NS difference seen vanilla flavoured Nic-EC compared to control.<br><br>Interpretation: Vaping nicotine EC may induce oxidative stress in lung in a flavour-specific manner.			
Caruso et al., 2022;[45] Italy	Independent funding organization(s); many (association with vape industry)	Acute; Single exposure	Human primary bronchial epithelial cells (NHBE)	PV/VG: Exposed to PG/VG at 1:1 ratio non nicotine EC.<br><br>PV/VG +Nic: Exposed to PG/VG 1:1 with tobacco flavoured EC containing 15 mg/L nicotine of 4 different brands.<br><br>Cig: Exposed to 1R6F cigarettes.<br>Air control.	Pulmonary oxidative stress; Not measured	ROS production:<br>NS production was seen in PG/VG exposure. Aerosol from PG/VG + Nic instead generated significant production of ROS ($p<0.05$) in proportion to the number of puffs.<br><br>Interpretation: Nicotine EC has induced significant oxidative stress in lung cells.	N/A	N/A	
Yogeswaran et al., 2022;[46] The US	Independent funding organization(s); none	Acute; Single exposure	BEAS-2B cell lines (lung epithelium)	Control group: exposed to air.<br><br>PG:VG: Exposed to PG:VG 50%:50%.	Pulmonary oxidative stress; Not measured	Levels of acellular ROS generation:<br>Were significantly higher than control ($p<0.05$) in exposure to PG:VG solution, PG:VG nicotine + WS-23, PG: VG + WS-3.<br>NS difference in acellular ROS	N/A	N/A	

				Nic-EC: Exposed to PG:VG 50%:50% and 5% nicotine.<br><br>PG:VG + cooling agent: exposed to PG:VG 50%:50% and 3% WS-23 or 3% WS-3.<br><br>Nic-EC + cooling agent: Exposed to PG:VG 50%:50% and 5% nicotine and 3% WS-23 or 3% WS-3.		level between e-liquid base solution (PG:VG), with and without nicotine, and addition of WS-23 nor WS-3 to it.<br><br>Levels of cellular ROS generation:<br>Were significantly higher in all groups compared to control ($p<0.05$).<br>NS difference in cellular ROS level between e-liquid base solution (PG:VG), with and without nicotine, and addition of WS-23 nor WS-3 to it.<br><br>Interpretation: EC exposure increases risk of oxidative stress irrespective of nicotine content and cooling agent.			
Park et al., 2021;[42] US	Independent funding organization(s); none	Acute; Single exposure	Primary normal Human Bronchial Epithelial cells (NHBE)	Control: water/medium: 2% v/v.<br><br>Nic-EC smoke solution: prepared from Blu vivid vanilla flavored 2.8% EC<br>Exposure given for 6 or 24 h.	Lung cancer susceptibility; not measured	Exposure to EC smoke solution disrupted biological pathways involving ribosomes and protein biogenesis in NHBE cells. Protein synthesis assay revealed that at 24 h, protein synthesis was significantly decreased by 88% and 23% at 2 and 5 ppm, respectively, compared to control ($p<0.05$). At 48 h, protein synthesis was significantly decreased by 79%, 74%, and 18% at 1, 2, and 5 ppm, respectively ($p<0.05$).<br><br>Interpretation: These data	N/A	N/A	

						indicate that exposure to nicotine EC may disrupt protein biogenesis in lung epithelium-increasing susceptibility to carcinogenic gene expression.			
Wang et al., 2022;[37] China	Not specified; none	Acute; 48hrs	16HBE cells	Control: exposed to air.<br><br>t-Cig CS: Exposed to condensate solutions prepared from traditional Cig.<br><br>EC (tobacco) CS: Exposed to condensate solutions prepared from tobacco flavored EC.<br><br>EC (menthol) CS: Exposed to condensate solutions prepared from menthol flavored EC.	Lung cancer; irreversible	Cell viability:<br>NS effect was seen EC (tobacco) CS and EC (menthol) CS groups, even when the concentration of nicotine reached 100 µg/mL.<br><br>Proteomic analysis:<br>Differential expressed exosomal proteins (DEEPs) were less expressed in both EC (tobacco) CS and EC (menthol) CS groups compared to t-Cig CS group. Among them, there were 4 DEEPs related to cancer found in EC (tobacco) CS and 3 found in EC (menthol) CS group compared to 14 DEEPs found in t-Cig CS group.<br><br>Interpretation: Acute exposure to EC did not significantly increased cancer related exosomal proteins in lung cells.	N/A	N/A	
Komura et al., 2022;[25] Japan	Independent funding organization(s); none	Acute; 4 hours	Primary human small airway epithelial cells (SAECs) from healthy donors and COPD-SAECs from	Control SAECs (PG:VG 0:0);<br><br>PG-treated SAECs (1-4% PG);<br><br>Gly-treated	Lung cancer; irreversible	Phosphorylated histone yH2AX (marker of DNA damage): Increased in SAECs exposed to 4% PG (p<0.0001) and was significantly greater in COPD-SAECs.<br><br>Cell cycle and apoptosis (caspase 3/7): An increased proportion of cells in the G1	N/A	N/A	

			COPD patients	SAECs (1-4% VG);<br><br>COPD-PG (1-4% PG) treated SAECs.		phase (p<0.0001) and increased p21 expression (p=0.0005) and increased caspase 3/7-activated cells (p=0.0054) were observed on exposure to 4% PG. The ratio of yH2AX-positive and caspase 3/7-positive cells was significantly higher in COPD-SAECs compared to that in control cells after exposure to 4% PG.<br><br>Interpretation: 4% PG can cause significant airway epithelial DNA damage, p21 expression and apoptosis, increasing risk of lung cancer. The effect was significantly higher in samples from people diagnosed with COPD.			
Trifunovic et al., 2022;[35] Croatia	Independent funding organization(s); none	Acute (Single exposure)	V79 lung fibroblasts	Control.<br><br>Positive Control.<br><br>PG/VG 'Virginia Tobacco' flavour.<br><br>Nic-EC 'Virginia Tobacco' flavour (250 µg/mL of nicotine).<br><br>Nicotine solution (250 µg/mL of	Lung mutagenicity; Irreversible	Mutagenicity: EC liquid showed no mutagenic or genotoxic effect, however it negatively impacted metabolic cooperation between V79 cells.<br><br>Gene expression:<br>Treatments with e-cigarette liquids, both PG/VG and Nic-EC, significantly increased number of 6-TG resistant colonies in V79 cells (p<0.01 for both), indicating impaired intercellular communication.<br><br>Protein production:<br>The untreated cells had the highest number of different proteins, approximately 10% more than pure nicotine	N/A	N/A	

				nicotine):<br>E-liquid delivered in sub cytotoxic concentration		treatment, 15% more than PG/VG and 23% more than Nic-EC. Increased presence of post-translational modifications, including carbonylation and direct oxidative modifications, was observed following e-liquid exposure.<br><br>Overall: Impairment of metabolic function and significant proteome and post translational modification alterations occurred in lung fibroblasts treated with e-liquid			
Molony et al. 12, 2023;[27] US	Independent funding organization; none	Acute; Single exposure (24 hrs)	Grade IV human TCCSUP bladder cancer cell line and SV-HUC (uroepithelium) cells	TCCSUP BC cells were either treated with-<br><br>Cig smoke extract (CSE) 1% concentration,<br><br>Unflavored e-liquid (UEL) 6% concentration,<br><br>Menthol-flavored e-liquid (MEL) 2% concentration,<br><br>Untreated.	Bladder cancer; irreversible	EC-treated bladder cancer-derived extracellular vesicles (BCEVs) were able to promote oxidative stress (ROS levels), inflammatory signaling (IL-6, MCP-1), and DNA damage, apoptosis (by CSE) and necrosis (by MEL) in recipient SV-HUC urothelial cells compared to untreated group ($p<0.05$). menthol E-liquid (MEL)-induced BCEVs significantly increased rates of malignant urothelial cell transformation ($P<0.0001$).<br><br>Interpretation: EC may increase risk of bladder cancer incidence or recurrence.	N/A	N/A	
Tsai et al., 2021;[36] US	Independent funding	Acute; Single exposure	N=201 samples of lung	LUSC samples: Smoking onset	Lung squamous cell carcinoma/	Differential expression analysis for eRNAs:<br>16 key eRNAs were detected	N/A	N/A	

	organization; none		squamous cell carcinoma (LUSC); lung epithelium samples from 10 donors	LUSC samples (n=129),<br><br>Non-smoking onset LUSC samples (n=18),<br><br>LUSC Adjacent normal tissue samples (n=49).<br><br>EC samples: Small airway epitheliums and alveolar macrophages-before inhalation of EC (n=10), after inhalation of EC (n=10). None of them were former smokers.	lung cancer; irreversible	which were highly associated with lung squamous cell carcinoma pathogenesis through chromosomal alterations and reduced CpG site DNA methylation. These key eRNAs ultimately upregulate the expression of oncogenic eRNAs and downregulate the expression of tumor-suppressing eRNAs.<br><br>EC samples had large eRNA expression upregulation after EC inhalation, which corresponded to the upregulation of the 16 key eRNAs.<br><br>Interpretation: EC aerosol may increase expression of key eRNAs which are involved in lung squamous cell carcinoma pathogenesis and reduce patient survival.			
Sun et al., 2022;[33] US	Independent funding organization; none	Acute, Short to medium term;<br>24 hrs, 1-4 weeks	Human lung bronchial epithelial BEAS-2B cell	0, 500, and 750 uM of nicotine for 24 h OR<br>0, 10, 25, and 50 uM for 1-4 weeks.	Cell transformation and potential carcinogenesis in lung; irreversible	Stem-loop binding protein (SLBP) protein levels were reduced significantly following 500 and 750mM nicotine exposure for 24 hrs or 50uM nicotine for 4 weeks as compared with control ($p<0.05$). Exposure to750mM of nicotine for 24 h or 50uM nicotine for 4 weeks results increased H3.1 mRNA with poly(A) tail, and an increase in H3 protein compared	N/A	N/A	

						to control (p<0.05).<br><br>Interpretation: High concentration of nicotine EC exposure increases risk of cell transformation and potential carcinogenesis in lung.			
Robin et al., 2022;[31] US	Independent funding organization; none	Acute; Single exposure	Ca9-22 human oral squamous carcinoma cells and Cal27 human tongue squamous carcinoma cells	Red Hot: Exposed to 'Red Hot' flavored EC vapor extract.<br><br>Red Hot + Nic: Exposed to 'Red Hot' flavored EC vapor extract with 6 mg/ml nicotine.<br><br>Apple: Exposed to 'Green Apple' flavored EC vapor extract.<br><br>Apple + Nic: Exposed to 'Green Apple' flavored EC vapor extract with 6 mg/ml nicotine.<br><br>Untreated Control: medium alone.	Oral squamous cell carcinoma; irreversible	Inflammatory markers: Elevated NF-kB, TNF-α, ERK, JNK, MMP-13 and cell invasion was detected by Ca9-22 cells treated with Apple+ Nic. Increased TNF-α and JNK level was detected by Ca9-22 cells treated with Red Hot+ Nic. Increased TNF-α and JNK were detected by Cal27 cells treated with both Apple + Nic and Red Hot+ Nic.<br><br>Interpretation: EC flavoring and nicotine causes differential cell invasion and inflammatory effect- initial step in cancerous invasion.	N/A	N/A	
Kwon et al., 2021;[26]	Independent funding	Acute; Single	Patient derived brain	E-liquid: exposed to	Brain tumour growth	E-liquid significant increased pEGFR and pERK expression	N/A	N/A	

South Korea	organization; none	exposure (24 hours)	tumour CSC2 cell line	19.7 ng/ml, 39.5 ng/ml, and 79.1 ng/ml nicotine concentration e-liquid mixture in culture media for 24 hours.<br><br>Vehicle: exposed to PG mixture in culture media for 24 hours.	(Glioblastoma Multiforme/GBM); irreversible	compared to vehicle (p<0.05); and significantly increased CSC2 cell growth rate (p<0.05) and self-renewal capacity of brain cancer stem cells (p<0.05) compared to vehicle.<br><br>Interpretation: E-liquid activates pEGFR and pERK, leading to accelerated brain tumor growth (GBM) and poor prognosis.			
de Lima et al., 2023;[18] Canada	Independent funding organization; none	Acute; Single exposure (72 hours)	Normal oral epithelium cell lines (NOE and HMK), oral squamous cell carcinoma (OSCC) human cell lines (CAL27 and HSC3), mouse oral cancer cell line (AT84)	E-liquid: included 1 mg/ml nicotine and 30%/70% PG/VG.<br><br>E-liquid was directly applied on the cell lines for 72 hours.	Oral squamous cell carcinoma; irreversible	Cell viability:<br>Significantly (p<0.001) increased cell death was observed in 1% e-liquid concentration in normal cells and 10% e-liquid concentration for OSCC cells compared to control, indicating increased sustainability for cancer cells.<br><br>Cell growth:<br>OSCC cell lines were able to survive after E-liquid treatment and exhibited anchorage-independent growth.<br><br>Epithelial to mesenchymal transition:<br>Both 0.1% and 1% e-liquid induced significant decrease in epithelial markers (E-cadherin) and increase in mesenchymal proteins (B-catenin and vimentin) in OSCC cell lines. RT-PCR showed that e-liquid	N/A	N/A	

						treatment significantly altered gene expression for CDH1, CTNNB1 and VIM in both normal cell and OSCC cell lines.<br><br>Interpretation: Direct acute exposure to e-liquid contributed to the tumorigenesis process in normal oral epithelium cells and promote growth of OSCC cells.			
Tellez et al, 2023;[34] US	Independent funding organization; none	Short to medium term (12 weeks)	human bronchial epithelial cell (HBEC2, HBEC4, HBEC6) lines	Control: Exposed to filtered air.<br><br>Unflavored (UF): Exposed to aerosol of unflavored nicotine EC.<br><br>Fruit EC (BP): Exposed to aerosol of Blue Pucker or fruit flavored nicotine EC.<br><br>Tobacco EC (JT): Exposed to aerosol of Jamestown or tobacco flavored nicotine EC.<br><br>Mardi Gras (MG): Exposed to aerosol of	Lung cancer; irreversible	Oxidative stress: was significant ($p<0.05$) in HBEC4 and HBEC6 cell lines on exposure to UF, BP, JT, and MG compared to control.<br><br>Cell transformation: was induced by UF, BP and JT aerosols in HBEC6 cell lines, but not by MG. However, the transformation was 3-fold lower than that seen in Cig exposure.<br><br>DNA damage: NS DNA damage was seen on exposure to UF or other 3 flavored EC.<br><br>Gene expression and modification: Transformation by EC induced alterations in canonical pathways implicated in lung cancer that included axonal guidance and NRF2.<br><br>Interpretation: EC exposure might cause cellular changes which predisposes individuals	N/A	N/A	

				Mardi Gras or fruit flavored nicotine EC.<br><br>Cig: Exposed to Kentucky reference Cig.<br><br>E-liquid contained 30%/70% PG/VG with free-base nicotine 1.2%. Exposure was given for 20 mins per day for 12 weeks.		to lung cancer. There were NS difference between unflavored and flavored EC exposure.			
Jeon et al., 2023;[22] US	Independent funding organization; none	Acute; (24 hrs and 7 days)	Human primary small airway epithelial cells (SAEC)	Custom built ENDS aerosol generation system was used to generate aerosols from JUUL pod-type ENDS using tobacco-flavored e-liquid of 5% nicotine.<br><br>Blank sample,<br><br>Negative Control: No EC exposure,<br><br>Puff fraction (1-50):	DNA damage (lung cancer); irreversible	ROS generation:<br>After both 24 hr, and 7 days exposure to puff fraction (101-150), there was significantly increased level of ROS compared to negative control ($p<0.01$) and puff fraction (1-50) ($p<0.01$).<br><br>Total glutathione level:<br>Significantly decreased following exposure to puff fraction (101-150) compared to negative control ($p<0.05$) at 24 hr and 7 days, and following exposure to puff fraction (1-50) compared to negative control at 7 days ($p<0.01$).<br><br>Cell viability:<br>Significantly decreased following exposure to puff	N/A	N/A	

				exposed at puff fraction 1-50 for 24 hrs and 7 days,<br><br>Puff fraction (101-150): exposed at puff fraction 101-150 for 24 hrs and 7 days,		fraction (101-150) compared to negative control ($p<0.05$) at 7 days, but NS reduction was seen following exposure to puff fraction (1-50).<br><br>Single stranded DNA damage: Significantly increased following exposure to puff fraction (101-150) compared to puff fraction (1-50) at 24 hrs. ($p<0.01$), and compared to both negative control and puff fraction (1-50) at 7 days ($p<0.0001$ and $p<0.01$ respectively).<br>Significantly increased following exposure to puff fraction (1-50) compared to negative control at 7 days only ($p<0.05$).<br><br>Toxic metal in aerosols: Significantly ($p<0.05$) increased level of toxic metals-chromium, copper, and lead were found in puff fraction (101-150).<br><br>Interpretation: Acute exposure to EC was associated with significant reduction in cell viability and increase in toxic metal production, oxidative stress and DNA damage during puff fraction 101-150.			
Zarcone et al., 2023;[39] France	Independent funding organization; None	Acute (single exposure)	human bronchial epithelial cell model	Control: negative control (air)	Genotoxicity, DNA damage (lung cancer); irreversible	Cell viability:<br>NS effect was seen on EC exposures.	N/A	N/A	

			(BEAS 2B cell line)	and positive control.<br><br>EC: exposed to tobacco flavored 65%/35% PG/VG and 16 mg/mL nicotine containing EC aerosol.<br><br>HTP: Exposed to IQOS.<br><br>Cig: Exposed to 3R4F research Cig.<br><br>EC aerosol exposure was given through a 3rd generation EC device in 18W and 30W settings.		Oxidative stress:<br>NS difference in Nrf2 antioxidative activity was seen between EC exposure and negative control. Expression of heme oxygenase 1 (HMOX1) protein and NAD(P)H Quinone Dehydrogenase 1 (NQO1) protein increased to a small extent by EC ($p < 0.05$) compared to negative control.<br><br>DNA damage and chromosomal aberrations:<br>NS difference was seen between EC exposure and negative control.<br><br>DNA methylation and histone modulations:<br>NS difference was seen between EC exposure and negative control.<br><br>Interpretation: NS risk of genotoxicity or lung cancer was seen on EC exposure.			
Effah et al., 2023;[48] UK	Independent funding organization; none	Acute (single exposure)	Human bronchial epithelial cells (HBEC-3KT)	Control: media only control,<br><br>PG/VG alone: exposed to non-flavored 60%/40% PG/VG containing e-liquids at 0.25%, 0.5% and 1% (v/v),	Lung oxidative stress; Not measured	Cell viability:<br>was significantly reduced in Cinnamon flavored PG/VG exposure compared to PG/VG alone and control (p<0.0001) in a dose-dependent manner.<br><br>ROS production:<br>Significantly increased in hazelnut and vanilla tobacco flavored PG/VG exposure compared to PG/VG alone and	N/A	N/A	

				Flavored PG/VG: Exposed to 15categories of flavored 60%/40% PG/VG containing e-liquids at 0.25%, 0.5% and 1% (v/v).		control (p<0.0001) in 1% (v/v).<br><br>Mitochondrial function: was significantly reduced in Cinnamon, hazelnut, and vanilla tobacco flavored PG/VG exposure compared to PG/VG alone and control (p<0.0001) in a dose-dependent manner.<br><br>Interpretation: Cinnamon, hazelnut and vanilla tobacco flavored non-nicotine EC exposure increased cytotoxicity and oxidative stress.			
Roxlau et al., 2023;[43] Germany	Independent funding organization(s); none	Acute (Single exposure)	Human bronchial epithelial cells (HBCE), human pulmonary arterial smooth muscle cells (hPASMCs), mouse pulmonary arterial smooth muscle cells (hPASMCs), mouse alveolar type II (mATII) cells.	Control: Exposed to filtered air.<br><br>PG/VG extract: exposed to nicotine free EC vapour extract prepared from 60%/30% PG/VG containing e-liquid.<br><br>Nic-EC extract: exposed to EC vapour extract prepared from 60%/30% PG/VG and 18mg/mL nicotine	Lung cancer susceptibility; not measured	Cell proliferation of hPASMCs and mPASMCs: was significantly (p<0.05) decreased in Nic-EC extract exposure compared to control. NS difference was seen between PG/VG extract and control exposure.<br><br>Metabolic activity: significantly decreased (p<0.05) in mATII, HBEC and hPASMCs, but not in mPASMCs on exposure to Nic-EC extract compared to control.<br><br>Interpretation: Nicotine containing EC vapor reduced cell proliferation and metabolic activity in lung parenchyma-indicating lower susceptibility to carcinogenesis.	N/A	N/A	

				containing e-liquid.<br><br>Cig extract: exposed to smoke of one 3R4F Cig.					
Begum et al., 2023;[47] US	Independent funding organization(s); none	Acute (24hrs)	Human alveolar epithelial cells (A549) cells	Control: Exposed to filtered air.<br><br>PG/VG: Exposed to non-nicotine tobacco-flavored (TF) EC vapor condensate (E CVC) containing 35%/75% PG/VG.<br><br>Nic-EC: Exposed to tobacco-flavored EC vapor condensate containing 35%/75% PG/VG with nicotine (6 mg/ml).	Lung oxidative stress; Not measured	ROS production: Significantly increased ($p<0.01$) in both PG/VG and Nic-EC exposure compared to control.<br><br>Oxidative stress: mRNA and protein expression of several NOX subunits and antioxidant enzymes significantly increased ($p<0.05$) in both PG/VG and Nic-EC exposure compared to control.<br><br>Interpretation: EC exposure both with and without nicotine caused significant oxidative stress in lung.	N/A	N/A	
**Animal studies**									
Alzoubi et al., 2022;[49] Jordan	Independent funding organization(s); none	Short to medium term; 1, 2 or 4 weeks	N=24; 6-8 weeks male mice (Balb/c)	Control (room air).<br><br>PG/VG (80:20 PG:VG): for 3	Lung oxidative stress; Not measured	Oxidative stress biomarkers: Significant decrease in GSH/GSSG ratio after 2 and 4 weeks and decrease in catalase and increase in thiobarbituric	Sex: male mice (n=24)	Same as study findings of entire population.	

				hours per day, 5 days per week from gestational day (GD) 5-20, excluding GDs 9,10,16, and 17.<br><br>Nic-EC (80:20 PG:VG + 5 or 10 mg/ml nicotine): 3 hours per day, 5 days per week from gestational day 5-20, excluding GDs 9,10,16, and 17.		acid reactive substances levels after 4 weeks of exposure.<br><br>Interpretation: Nicotine EC use caused oxidative stress in lung.			
Been et al., 2022;[50] Canada	Independent funding organization(s); none	Acute (3 days)	Male and female C57BL/6J mice; 8-12 weeks old	"Mango flavoured JUUL puff.<br><br>Mint flavoured JUUL puff.<br><br>Virginia tobacco flavoured JUUL puff.<br><br>PG/VG: exposed to control liquid made of 30:70 ratio of PG and VG.	Lung oxidative stress; Not measured	Oxidative markers: Significant increase in blood biomarkers of oxidative stress (8-OHdG by mint flavoured JUUL, MDA by tobacco flavoured JUUL) compared to control.<br>At 4 puffs/min exposure, Significant increase in blood biomarkers of oxidative stress (8-OHdG by mint flavoured JUUL, MDA by tobacco flavoured JUUL) and oxidative stress gene expression (Sod2 by mint flavoured JULL, Nrf2 by tobacco flavoured JUUL) compared to control.<br><br>Interpretation: Vaping nicotine	N/A	N/A	

				Control: Exposed to air.<br><br>JUUL contained 59 mg/ml nicotine. Mice were exposed for 20 mins per day for 3 consecutive days.		EC was associated with oxidative stress in lung.			
Hassan and El-Wafaey, 2022;[51] Egypt	None; none	Short-to-medium term (4 weeks)	N=20; adult male Wistar albino rat	Control Group: n=10, exposed to fresh air.<br><br>EC group: n=10. exposed to 1 ml/day e-liquid aerosol.<br><br>Exposure was given for 5 consecutive days a week for 4 consecutive weeks	Lung oxidative stress; Not measured	Oxidative stress biomarkers- malondialdehyde (MDA) levels, total antioxidant capacity (TAC):<br>EC group had a significant elevation of MDA and suppression in TAC levels in comparison with the control group ($p<0.001$) in both larynx and lung. Increase of α-SMA and P53 immuno-expression was also noted in EC group.<br><br>Interpretation: Exposure to e-liquid with nicotine induced significant oxidative stress in lung.	Sex: male rats (n=20)	Same as study findings of entire population.	
Platel et al., 2022;[29] France	Independent funding organization; none	Acute, Short to medium term;<br>4 days,<br>3 months and 6 months	N=30; 9 weeks old male BALB/c mice	Negative Control: Exposed to air (n=5).<br><br>Cigarette: Exposed to 3R4F cigarette smoke (n=5).<br><br>E-cig aerosol	DNA damage (lung and liver); irreversible	Acute exposure:<br>NS increase in DNA strand breaks was observed following any EC exposure. Exposures to Modbox 30W EC aerosol emissions for 60min and 90min induced a significant increase in 8-OHdG levels or oxidative stress compared to the control ($p<0.05$ for both).	Sex: Male (n=30)	Same as study findings of entire population	

				Modbox 18 W (n=5) and 30W (n=5).<br><br>Positive Control: Exposed to methyl methanesulfonate<br><br>All groups were given exposure in acute (4 days), short-to-medium term exposure (5 days a week for (3 months and 6 months).		Short-to-medium term exposure:<br>At 3 months NS increase in the level of DNA strand breaks was observed for both EC exposure. After exposure to Modbox 30W aerosol, there was a significant increase in 8-OHdG level compared to control ($p<0.05$) while an exposure to Modbox 18W emissions induced NS change.<br>At 6 months, statistically significant increases ($p<0.05$) in DNA strand breaks were observed for Modbox 30W in the liver and in the lung. Exposure to Mb30W aerosol induced a statistically significant increase in 8-OHDG level ($p<0.05$).<br><br>Interpretation: Only the high-power EC exposure induced DNA damage in the lung and the liver of exposed mice following short-to-medium term exposure.			
Sun et al., 2022;[33] US	Independent funding organization; none	Short to medium term; 3 months	N=28; 8 weeks old female A/J mice	Baseon exposure-<br>FA: filtered air (n=8).<br><br>PG/VG (50:50) (n=8).<br><br>PG/VG + Nicotine (50:50 + 36mg/ml)	Cell transformation and potential carcinogenesis in lung; irreversible	Both protein and mRNA levels of Stem-loop binding protein (SLBP) were decreased in the PG/VG + nicotine group compared to FA group ($p<0.05$), but not in the PG/VG group.<br>The level of polyadenylated mRNA expression was also increased in lung tissues of mice exposed to PG/VG + nicotine, but not to only PG/VG.	N/A	N/A	

				(n=12).<br><br>EC aerosol was delivered for 4 h per day, 5 days a week, for 3 months		Nicotine-induced SLBP depletion is reversed by an inhibitor of a7-nAChR or siRNA specific for a7-nAChR, indicating a nAChR-dependent reduction of SLBP by nicotine. Moreover, PI3K/AKT pathway is activated by nicotine exposure and CK2 and probably CDK1 were involved in nicotine-induced SLBP depletion.<br><br>Interpretation: Nicotine EC exposure increases risk of cell transformation and potential carcinogenesis in lung.			
Kwon et al., 2021;[26] South Korea	Independent funding organization; none	Short to medium term; 20-41 days	N=10; 5 weeks old female BALB/c nude mice	E-liquid: (n=4) Patient derived brain tumour CSC2 cell lines were injected in mice brain followed by 103μg/ml nicotine e-liquid was fed to mice daily.<br><br>Vehicle: (n=4) CSC2 cell lines were injected in mice brain followed by PG solution was fed to mice daily.	Brain tumour growth (Glioblastoma Multiforme/GBM); irreversible	E-liquid accelerated tumour growth on MRI and increased activation of pEGFR and pERK ($p<0.05$) on histological staining compared to control.<br><br>Interpretation: E-liquid activates pEGFR and pERK, leading to accelerated brain tumor growth (GBM) and poor prognosis.	Sex: female mice (n=10)	Same as study findings of entire population	

Muthumalage and Rahman, 2023;[28] US	Independent funding organization(s); none	Acute (3 days)	N=16-20; 8–10-week-old male and female e C57BL/6J and BALB/cJ mice	Control: Exposed to air.<br><br>PG/VG: Exposed to EC aerosol containing PG/VG at 1:1 ratio.<br><br>Menthol 0mg. or 6 mg. Nic: Exposed to 0mg/ml or 6 mg/ml nicotine menthol flavored EC aerosol,<br><br>Tobacco 0mg. or 6 mg. Nic: Exposed to 0mg/ml or 6 mg/ml nicotine tobacco flavored EC aerosol,<br><br>EC exposure was given by 3rd generation ENDS for 2 hrs for 3 consecutive days.	Lung genotoxicity/ DNA damage; not measured	Genotoxicity/DNA damage markers in lung tissue: Significantly increased genotoxicity markers ($p<0.05$) including the primary negative regulator of p53, were noted in menthol 0mg. Nic and tobacco 6mg. Nic exposure compared to control.<br><br>Interpretation: Acute exposure to flavored EC, both with and without nicotine significantly increased risk of DNA damage in lung.	N/A	N/A	
Daou et al., 2021;[52] Lebanon	Independent funding	Acute (7 days)	N=64; 4 months old FVB-Tg	Diabetic + EC (n=8) and non-diabetic + EC	Lung oxidative	ROS production: Significant increase in diabetic mice exposed to EC compared	Sex: female (n=64)	Same as study findings of	

	organization(s); none		mice (diabetic and non-diabetic) female mice	(n=8): Exposed to 18mg/ml nicotine EC.<br><br>Diabetic + HTP (n=8) and non-diabetic + HTP (n=8): Exposed to IQOS.<br><br>Diabetic + CS (n=8) and non-diabetic + CS (n=8): Exposed to 3R4F cigarettes, 9.4 mg tar, and 0.726 mg nicotine per Cig.<br><br>Diabetic + Control (n=8) and non-diabetic + Control (n=8): Exposed to air.	stress; Not measured	to non-diabetic+ EC mice (p<0.05), increase was higher in CS exposure compared to EC exposure.<br><br>Cellular apoptosis: Significant increase in diabetic mice exposed to EC compared to non-diabetic+ EC (p<0.05).<br><br>Interpretation: Diabetes (and other comorbidities) may exacerbate the effects of nicotine-containing e-cigarettes.		entire population.	

Abbreviation: CI, confidence interval; Cig, cigarette; COPD, chronic obstructive pulmonary disease; DNA, deoxyribonucleic acid; EHR, electronic health record; NH, Non-Hispanic; ENDS, electronic nicotine delivery system; EC, e-cigarette; GSH?GSSG, glutathione/oxidized glutathione ratio; OR, odds ratio; Nic, nicotine; N/A, not applicable; NS, not significant; pEGFR, phosphorylated epidermal growth factor receptor; pERK, phosphorylated Mitogen-activated protein kinase; PG, propylene glycol; US, the United States; UK, the United Kingdom; VG, vegetable glycerin;

Supplemental Material 2. Summary of studies retrieved from the KCL review for subgroup analysis on risk of cancer from exposure to e-cigarettes (n=2)

**Authors, Year; Country**	**Sub-group category**	**Sub-group description**	**Number of participants in sub-group**	**Type of exposure**	**Intervention/ Exposure**	**Health effect outcomes**	**Funding**	**Risk of Bias**
**Single sex animal studies**								
Pham et. al, 2022;[53] USA	Sex	Female 5-7 weeks old BALB/C mice	16 mice in air/EC (8 per group)	Short-to-medium term (6 weeks)	EC group: exposed to e-cig generated from e-liquid (24 mg/mL of nicotine dissolved in the mixture of PG and VG at a 1:1 ratio).<br><br>Air: exposed to air.<br><br>After 1 week of air or EC exposure in mice, breast cancer cells were injected orthotopically to establish breast cancer and/or lung metastasis.<br><br>EC exposure given as 70 mL puff volume, 1 puff/min, 2 h per day, 5 days per week for 6 weeks.	Tumor growth was significantly faster in EC than control.<br>Exposure associated with 100% tumor development in EC, only 33.3% in control.<br>Increase in proliferation by 15.74% and decrease in apoptosis (p = 0.0007) by 14.98%, in EC group.<br>Increased metastatic lung potential of mice injected with 4T07 cells.<br>Tumour nodules - 60.2% increase (p=0.024), 4T07 cells - 77.5% increase (p=0.036)<br>Significant increase in circulating monocytes in EC group.<br>Upregulation of the chemokine receptor, CCR5 (P value = 0.05), upward trend of CX3CR1 expression (p=0.08), number of F4/80-labeled resident monocytes increased by a factor of 4 and 2.5, respectively, in primary and metastatic sites of tumour following EC exposure.	Not Reported	N/A
Huynh et al., 2020;[54] USA	Sex	Four-week-old female NOD-SCID-Gamma (NSG) mice	Not specified	Short-to-medium term (4 weeks)	EC exposure: Exposing mice to EC (24 mg/mL nicotine) five days a week for 2 hours a day for 4 weeks<br><br>Control: exposed to air.	Lung colonization of breast cancer cells:<br>Lung localization of tumor cells in mice exposed to EC was clearly higher than that of the air controls. No prominent nodules on lung surfaces in either EC or air-exposed group<br><br>Double immunohistochemical stains showed that the level of positive GFP cells or tumor areas was very increased as compared to air controls. However, with Ki67 staining, human	Not Reported	N/A

						breast cancer proliferation after EC treatment had no significant changes.<br><br>Tumor apoptosis in lung colonized breast tumors of air-exposed mice was more notable than that of EC-exposed mice (cleaved caspase-3 stain).<br><br>Morphometric analysis indicates that tumor area in the EC treatment group was 2-fold greater than that of the air control group (P=0.0036) while Ki67 staining cells between the two groups were not different. Apoptotic GFP-positive cells in EC-exposed lung sections were 5-fold lower as compared to those of the air-exposed group		

Abbreviation: EC, e-cigarette; N/A, not applicable; PG, propylene glycol; VG, vegetable glycerin.

Supplemental Material 4. Quality assessment findings

Table 1. Risk of bias assessment of longitudinal observational studies with the Risk of Bias in Non-randomized Studies- of Exposure (ROBINS-E) tool.

Author and year	Overall based on B2/B3/B4	Domain 1	Domain 2	Domain 3	Domain 4	Domain 5	Domain 6	Domain 7	Overall
Goldberg Scott et al., 2023;[20] US	Needs further assessment	Low	Low	Low	Low	Low	Low	Low	**Low**
Chen et al., 2023;[17] US	Very high	-	-	-	-	-	-	-	**Very high**

Table 2. Risk of bias assessment of biomarker-based cross-sectional studies with the BIOCROSS tool.

Author and year	Item 1	Item 2	Item 3	Item 4	Item 5	Item 6	Item 7	Item 8	Item 9	Item 10	Overall
Guo et al., 2021;[21] US	2	1	0	0	1	2	1	2	1	1	**Moderate (11/20)**
Kamal and Shams, 2022;[23] Egypt	2	1	1	0	1	2	1	2	1	0	**Moderate (11/20)**
Richmond et al. 4, 2021;[30] UK	2	2	1	1	2	2	2	2	1	0	**Low (15/20)**
Song et al., 2023;[32] Germany	2	2	1	1	2	2	2	1	1	1	**Low (15/20)**

Abbreviation: NA= not applicable.

Table 3. Risk of bias assessment of non-biomarker based cross-sectional studies and case reports with the Joanna Briggs Institute (JBI) critical appraisal tools.

Author and year	Item 1	Item 2	Item 3	Item 4	Item 5	Item 6	Item 7	Item 8	Overall
**Cross-sectional studies**									
Dugan et al., 2021;[19] US	Y	Y	N	Y	N	Y	N	Y	**Moderate (5/8)**
Wharram et al., 2023;[38] US	Y	Y	Y	Y	Y	Y	Y	Y	**Low (8/8)**
Catto et al., 2023;[16] Netherlands	Y	Y	Y	Y	N	N	Y	Y	**Low (6/8)**
Austin-Datta et al., 2023;[14] US	Y	Y	N	N	Y	Y	N	Y	**Moderate (5/8)**
Kim and Keegan, 2022;[24] US	N	Y	Y	Y	Y	Y	Y	Y	**Low (7/8)**
**Case reports**									
Ballenberger et al., 2022;[15] US	N	N	Y	Y	Y	N	N	Y	**Moderate (4/8)**

Abbreviation: Y= yes, N=No, UC= unclear, NA= not applicable.

Supplemental Material 5. Validation of the KCL review

We aimed to replicate the study selection and data extraction process of the KCL review[1] and the results are presented below.

Validating the Study Selection

Of the 1000 search results that we retrieved from the MEDLINE database using the KCL review search strategy,[1] 2 duplicates were removed, and 891 studies were considered irrelevant. 107 studies therefore underwent full text screening, of which 59 studies were excluded and 48 studies were included.[2–49] Fig 1. summarises the selection criteria process. Out of the 59 excluded studies, only 1 was originally included in the KCL review.[50] We excluded this study[50] as it was reported as published after July 2021 which is beyond the cut off time for the KCL review search period. However, clearly it was accessible to the KCL authors at the time. Moreover, the study mainly evaluated burn and accidental injury from e-cigarette use. Given the fact that it is not a main health outcome for our review, this does not have a meaningful difference to our study selection compared to that of the KCL review.

We included a total of 48 studies, of which 10 studies were not included in the KCL review.[5,7,9–12,20,36,38,40] The majority of these differences were cell/*in vitro* studies or animal studies. 1 study was on coronavirus exacerbation in a murine model, however, coronavirus was not a focused health outcome in the KCL review.[7] 1 animal[5] and 1 cell/*in vitro* study[10] were on ACE-2 expression. 3 studies were cell/*in vitro* studies on toxicity and/or inflammation.[11,12,20] One study was on the impact of e-cigarettes on free radicals.[9] 1 cell/*in vitro* [36] and 1 human study[38] looked at oxidative stress biomarkers, however the human study was published on June 30th, 2021. 1 study investigated the effects of e-cigarette on the blood-brain barrier.[40]

Overall, given that discrepancies lie around mainly cell/*in vitro* studies and smaller topics, we do not consider there to be major differences between our study selection and the study selection conducted by the KCL review.[1]

Validating the Data Extraction

Of the 40 articles randomly selected from the KCL review,[1] there were 8 biomarkers of nicotine and toxicant exposure studies,[8,48,52–57] 16 biomarkers on potential harm to health studies,[44,54,58–71] 11 animal studies,[13,25,26,33,45,47,49,72–75] 4 cell studies[76–79] and 2 poisoning, fires and burns studies.[80,81] Overall, the findings in the extracted data matched the data from the KCL review,[1] and any differences were small and did not impact the overall interpretation of the studies.

Detailed data on the validation of the KCL review are available upon request to the corresponding author.

References

1. Nicotine vaping in England: an evidence update including health risks and perceptions, September 2022. Published online 2022.
2. Jude J, Hiller H, Miller J. Melon with a Twist: A Case of Nicotine Overdose After Ingestion and Aspiration of Vape Liquid. *Mil Med*. Published online October 10, 2020:usaa331. doi:10.1093/milmed/usaa331
3. Maloney S, Eversole A, Crabtree M, Soule E, Eissenberg T, Breland A. Acute effects of JUUL and IQOS in cigarette smokers. *Tob Control*. Published online February 10, 2020:tobaccocontrol-2019-055475. doi:10.1136/tobaccocontrol-2019-055475
4. Cirillo S, Vivarelli F, Turrini E, et al. The customizable e-cigarette resistance influences toxicological outcomes: lung degeneration, inflammation and oxidative stress-induced in a rat model. *Toxicol Sci*. Published online August 6, 2019:kfz176. doi:10.1093/toxsci/kfz176
5. Masso-Silva JA, Moshensky A, Shin J, et al. Chronic E-Cigarette Aerosol Inhalation Alters the Immune State of the Lungs and Increases ACE2 Expression, Raising Concern for Altered Response and Susceptibility to SARS-CoV-2. *Front Physiol*. 2021;12:649604. doi:10.3389/fphys.2021.649604
6. Molino AR, Jerry-Fluker J, Atkinson MA, Furth SL, Warady BA, Ng DK. The association of alcohol, cigarette, e-cigarette, and marijuana use with disease severity in adolescents and young adults with pediatric chronic kidney disease. *Pediatr Nephrol*. 2021;36(8):2493-2497. doi:10.1007/s00467-021-05044-5
7. Sivaraman V, Parker D, Zhang R, Jones MM, Onyenwoke RU. Vaping Exacerbates Coronavirus-Related Pulmonary Infection in a Murine Model. *Front Physiol*. 2021;12:634839. doi:10.3389/fphys.2021.634839
8. McEwan M, Gale N, Ebajemito JK, et al. A randomized controlled study in healthy participants to explore the exposure continuum when smokers switch to a tobacco heating product or an E-cigarette relative to cessation. *Toxicol Rep*. 2021;8:994-1001. doi:10.1016/j.toxrep.2021.05.003
9. Suryadinata RV, Wirjatmadi B, Lorensia A. The time pattern of selenomethionine administration in preventing free radicals due to exposure to electric cigarette smoke. *J Public Health Res*. 2021;10(2):2232. doi:10.4081/jphr.2021.2232
10. McAlinden KD, Lu W, Ferdowsi PV, et al. Electronic Cigarette Aerosol Is Cytotoxic and Increases ACE2 Expression on Human Airway Epithelial Cells: Implications for SARS-CoV-2 (COVID-19). *J Clin Med*. 2021;10(5):1028. doi:10.3390/jcm10051028
11. Rickard BP, Ho H, Tiley JB, Jaspers I, Brouwer KLR. E-Cigarette Flavoring Chemicals Induce Cytotoxicity in HepG2 Cells. *ACS Omega*. 2021;6(10):6708-6713. doi:10.1021/acsomega.0c05639
12. De Martin S, Gabbia D, Bogialli S, et al. Refill liquids for electronic cigarettes display peculiar toxicity on human endothelial cells. *Toxicology Reports*. 2021;8:456-462. doi:10.1016/j.toxrep.2021.02.021
13. Sharma A, Lee J, Fonseca AG, et al. E-cigarettes compromise the gut barrier and trigger inflammation. *iScience*. 2021;24(2):102035. doi:10.1016/j.isci.2021.102035
14. Song SY, Na HG, Kwak SY, Choi YS, Bae CH, Kim YD. Changes in Mucin Production in Human Airway Epithelial Cells After Exposure to Electronic Cigarette Vapor With or Without Nicotine. *Clin Exp Otorhinolaryngol*. 2021;14(3):303-311. doi:10.21053/ceo.2020.01907
15. Rosenkilde Laursen K, Bonlokke JH, Bendstrup E, et al. An RCT of acute health effects in COPD-patients after passive vape exposure from e-cigarettes. *European Clinical Respiratory Journal*. 2020;8(1):1861580.
16. Alhaddad H, Wong W, Sari AT, Crotty Alexander LE, Sari Y. Effects of 3-Month Exposure to E-Cigarette Aerosols on Glutamatergic Receptors and Transporters in Mesolimbic Brain Regions of Female C57BL/6 Mice. *Toxics*. 2020;8(4):95. doi:10.3390/toxics8040095
17. Polosa R, Morjaria JB, Prosperini U, et al. COPD smokers who switched to e-cigarettes: health outcomes at 5-year follow up. *Ther Adv Chronic Dis*. 2020;11:2040622320961617. doi:10.1177/2040622320961617
18. Smith DM, Shahab L, Blount BC, et al. Differences in Exposure to Nicotine, Tobacco-Specific Nitrosamines, and Volatile Organic Compounds among Electronic Cigarette Users, Tobacco Smokers, and Dual Users from Three Countries. *Toxics*. 2020;8(4):88. doi:10.3390/toxics8040088
19. Makri OE, Pallikari A, Kagkelaris K, et al. The Acute Effects of Electronic Cigarette Vaping and Tobacco Cigarette Smoking on Choroidal Thickness in Young, Healthy, Habitual, Dual Smokers. *Toxics*. 2020;8(4):85. doi:10.3390/toxics8040085

20. Lucas JH, Muthumalage T, Wang Q, Friedman MR, Friedman AE, Rahman I. E-Liquid Containing a Mixture of Coconut, Vanilla, and Cookie Flavors Causes Cellular Senescence and Dysregulated Repair in Pulmonary Fibroblasts: Implications on Premature Aging. *Front Physiol*. 2020;11:924. doi:10.3389/fphys.2020.00924
21. Huynh D, Huang J, Le LTT, et al. Electronic cigarettes promotes the lung colonization of human breast cancer in NOD-SCID-Gamma mice. *Int J Clin Exp Pathol*. 2020;13(8):2075-2081.
22. Ramanathan G, Craver-Hoover B, Arechavala RJ, et al. E-Cigarette Exposure Decreases Bone Marrow Hematopoietic Progenitor Cells. *Cancers (Basel)*. 2020;12(8):2292. doi:10.3390/cancers12082292
23. Lan K, Zhang G, Liu L, et al. Electronic cigarette exposure on insulin sensitivity of ApoE gene knockout mice. *Tob Induc Dis*. 2020;18:68. doi:10.18332/tid/125399
24. Richmond SA, Pike I, Maguire JL, Macpherson A. E-cigarettes: A new hazard for children and adolescents. *Paediatr Child Health*. 2018;23(4):255-259. doi:10.1093/pch/pxx204
25. Wang Q, Sundar IK, Li D, et al. E-cigarette-induced pulmonary inflammation and dysregulated repair are mediated by nAChR α7 receptor: role of nAChR α7 in SARS-CoV-2 Covid-19 ACE2 receptor regulation. *Respir Res*. 2020;21(1):154. doi:10.1186/s12931-020-01396-y
26. Prasedya ES, Ambana Y, Martyasari NWR, Aprizal Y, Nurrijawati null, Sunarpi null. Short-term E-cigarette toxicity effects on brain cognitive memory functions and inflammatory responses in mice. *Toxicol Res*. 2020;36(3):267-273. doi:10.1007/s43188-019-00031-3
27. Wang B, Liu ST, Rostron B, Hayslett C. Burn injuries related to E-cigarettes reported to poison control centers in the United States, 2010–2019. *Inj Epidemiol*. 2020;7:36. doi:10.1186/s40621-020-00263-0
28. Serra R, Menicagli R, Marotta O. Free radical production in the smoking of e-cigarettes and their possible effects in human health. *International Journal of Preventive Medicine*. 2020;11(1). https://www.ncbi.nlm.nih.gov/pmc/articles/PMC7297425/
29. Hagarty S, Luo J. E-cigarette "Vape" Device Explosion Causing C Spine Fracture. *Plast Reconstr Surg Glob Open*. 2020;8(4):e2745. doi:10.1097/GOX.0000000000002745
30. Al-Hamoudi N, Alsahhaf A, Al Deeb M, Alrabiah M, Vohra F, Abduljabbar T. Effect of scaling and root planing on the expression of anti-inflammatory cytokines (IL-4, IL-9, IL-10, and IL-13) in the gingival crevicular fluid of electronic cigarette users and non-smokers with moderate chronic periodontitis. *J Periodontal Implant Sci*. 2020;50(2):74-82. doi:10.5051/jpis.2020.50.2.74
31. Gellatly S, Pavelka N, Crue T, et al. Nicotine-Free e-Cigarette Vapor Exposure Stimulates IL6 and Mucin Production in Human Primary Small Airway Epithelial Cells. *J Inflamm Res*. 2020;13:175-185. doi:10.2147/JIR.S244434
32. Sinha DK, Vishal, Kumar A, Khan M, Kumari R, Kesari M. Evaluation of tumor necrosis factor-alpha (TNF-α) and interleukin (IL)-1β levels among subjects vaping e-cigarettes and nonsmokers. *J Family Med Prim Care*. 2020;9(2):1072-1075. doi:10.4103/jfmpc.jfmpc_902_19
33. Wawryk-Gawda E, Chylińska-Wrzos P, K. Zarobkiewicz M, Chłapek K, Jodłowska-Jędrych B. Lung histomorphological alterations in rats exposed to cigarette smoke and electronic cigarette vapour. *Exp Ther Med*. 2020;19(4):2826-2832. doi:10.3892/etm.2020.8530
34. Pushalkar S, Paul B, Li Q, et al. Electronic Cigarette Aerosol Modulates the Oral Microbiome and Increases Risk of Infection. *iScience*. 2020;23(3):100884. doi:10.1016/j.isci.2020.100884
35. Rao P, Liu J, Springer ML. JUUL and Combusted Cigarettes Comparably Impair Endothelial Function. *Tob Regul Sci*. 2020;6(1):30-37. doi:10.18001/TRS.6.1.4
36. Wavreil FDM, Heggland SJ. Cinnamon-flavored electronic cigarette liquids and aerosols induce oxidative stress in human osteoblast-like MG-63 cells. *Toxicol Rep*. 2020;7:23-29. doi:10.1016/j.toxrep.2019.11.019
37. Andersen A, Reimer R, Dawes K, et al. DNA methylation differentiates smoking from vaping and non-combustible tobacco use. *Epigenetics*. 2022;17(2):178-190. doi:10.1080/15592294.2021.1890875
38. Gupta R, Lin Y, Luna K, et al. Electronic and Tobacco Cigarettes Alter Polyunsaturated Fatty Acids and Oxidative Biomarkers. *Circulation Research*. 2021;129(5):514-526. doi:10.1161/CIRCRESAHA.120.317828

39. Lallai V, Manca L, Fowler CD. E-cigarette vape and lung ACE2 expression: Implications for coronavirus vulnerability. *Environ Toxicol Pharmacol*. 2021;86:103656. doi:10.1016/j.etap.2021.103656
40. Kadry H, Noorani B, Bickel U, Abbruscato TJ, Cucullo L. Comparative assessment of in vitro BBB tight junction integrity following exposure to cigarette smoke and e-cigarette vapor: a quantitative evaluation of the protective effects of metformin using small-molecular-weight paracellular markers. *Fluids Barriers CNS*. 2021;18(1):28. doi:10.1186/s12987-021-00261-4
41. Lim SW, Zulkiflee AB. Objective assessment of nasal resistance among electronic cigarette users. *Journal of Laryngology & Otology*. Published online 2021:1-4.
42. Majid S, Keith RJ, Fetterman JL, et al. Lipid profiles in users of combustible and electronic cigarettes. *Vasc Med*. 2021;26(5):483-488. doi:10.1177/1358863X211009313
43. Jarrell ZR, Smith MR, He X, Orr M, Jones DP, Go YM. Firsthand and Secondhand Exposure Levels of Maltol-Flavored Electronic Nicotine Delivery System Vapors Disrupt Amino Acid Metabolism. *Toxicol Sci*. 2021;182(1):70-81. doi:10.1093/toxsci/kfab051
44. Phillips-Waller A, Przulj D, Smith KM, Pesola F, Hajek P. Nicotine delivery and user reactions to Juul EU (20 mg/ml) compared with Juul US (59 mg/ml), cigarettes and other e-cigarette products. *Psychopharmacology (Berl)*. 2021;238(3):825-831. doi:10.1007/s00213-020-05734-2
45. Wong ET, Szostak J, Titz B, et al. A 6-month inhalation toxicology study in Apoe-/- mice demonstrates substantially lower effects of e-vapor aerosol compared with cigarette smoke in the respiratory tract. *Arch Toxicol*. 2021;95(5):1805-1829. doi:10.1007/s00204-021-03020-4
46. Rudasingwa G, Kim Y, Lee C, Lee J, Kim S, Kim S. Comparison of Nicotine Dependence and Biomarker Levels among Traditional Cigarette, Heat-Not-Burn Cigarette, and Liquid E-Cigarette Users: Results from the Think Study. *Int J Environ Res Public Health*. 2021;18(9):4777. doi:10.3390/ijerph18094777
47. Henderson BJ, Cooper SY. Nicotine formulations impact reinforcement-related behaviors in a mouse model of vapor self-administration. *Drug Alcohol Depend*. 2021;224:108732. doi:10.1016/j.drugalcdep.2021.108732
48. Coleman SRM, Bunn JY, Nighbor TD, et al. Use of electronic nicotine delivery systems (ENDS) among U.S. women of reproductive age: Prevalence, reported reasons for use, and toxin exposure. *Prev Med*. 2021;152(Pt 2):106582. doi:10.1016/j.ypmed.2021.106582
49. Naidu V, Zeki AA, Sharma P. Sex differences in the induction of angiotensin converting enzyme 2 (ACE-2) in mouse lungs after e-cigarette vapor exposure and its relevance to COVID-19. *J Investig Med*. 2021;69(5):954-961. doi:10.1136/jim-2020-001768
50. Flores CE, Chestovich PJ, Saquib S, et al. Electronic Cigarette-Related Injuries Presenting to Five Large Burn Centers, 2015-2019. *J Burn Care Res*. 2021;42(6):1254-1260. doi:10.1093/jbcr/irab114
52. Boykan R, Goniewicz ML, Messina CR. Evidence of Nicotine Dependence in Adolescents Who Use Juul and Similar Pod Devices. *Int J Environ Res Public Health*. 2019;16(12).
53. Prokopowicz A, Sobczak A, Szuła-Chraplewska M, Ochota P, Kośmider L. Exposure to Cadmium and Lead in Cigarette Smokers Who Switched to Electronic Cigarettes. *Nicotine Tob Res*. 2019;21(9):1198-1205. doi:10.1093/ntr/nty161
54. Ikonomidis I, Katogiannis K, Kostelli G, et al. Effects of electronic cigarette on platelet and vascular function after four months of use. *Food Chem Toxicol*. 2020;141:111389. doi:10.1016/j.fct.2020.111389
55. Jain RB. Re-visiting serum cotinine concentrations among various types of smokers including cigarette only smokers: some new, previously unreported results. *Environ Sci Pollut Res Int*. 2021;28(3):3149-3161. doi:10.1007/s11356-020-10677-4
56. Jacob P, St Helen G, Yu L, et al. Biomarkers of Exposure for Dual Use of Electronic Cigarettes and Combustible Cigarettes: Nicotelline, NNAL, and Total Nicotine Equivalents. *Nicotine Tob Res*. 2020;22(7):1107-1113. doi:10.1093/ntr/ntz235
57. Cobb CO, Foulds J, Yen MS, et al. Effect of an electronic nicotine delivery system with 0, 8, or 36 mg/mL liquid nicotine versus a cigarette substitute on tobacco-related toxicant exposure: a four-arm, parallel-group, randomised, controlled trial. *The Lancet Respiratory Medicine*. 2021;9(8):840-850. doi:10.1016/S2213-2600(21)00022-9

58. Baldassarri SR, Hillmer AT, Anderson JM, et al. Use of Electronic Cigarettes Leads to Significant Beta2-Nicotinic Acetylcholine Receptor Occupancy: Evidence From a PET Imaging Study. *Nicotine Tob Res*. 2018;20(4):425-433. doi:10.1093/ntr/ntx091
59. Solingapuram Sai KK, Zuo Y, Rose JE, et al. Rapid Brain Nicotine Uptake from Electronic Cigarettes. *J Nucl Med*. 2020;61(6):928-930. doi:10.2967/jnumed.119.230748
60. Voos N, Kaiser L, Mahoney MC, et al. Randomized within-subject trial to evaluate smokers' initial perceptions, subjective effects and nicotine delivery across six vaporized nicotine products. *Addiction*. 2019;114(7):1236-1248. doi:10.1111/add.14602
61. O'Connell G, Pritchard JD, Prue C, et al. A randomised, open-label, cross-over clinical study to evaluate the pharmacokinetic profiles of cigarettes and e-cigarettes with nicotine salt formulations in US adult smokers. *Intern Emerg Med*. 2019;14(6):853-861. doi:10.1007/s11739-019-02025-3
62. Vohra F, Bukhari IA, Sheikh SA, Albaijan R, Naseem M. Comparison of self-rated oral symptoms and periodontal status among cigarette smokers and individuals using electronic nicotine delivery systems. *J Am Coll Health*. 2020;68(7):788-793. doi:10.1080/07448481.2019.1709476
63. Atuegwu NC, Perez MF, Oncken C, Thacker S, Mead EL, Mortensen EM. Association between Regular Electronic Nicotine Product Use and Self-reported Periodontal Disease Status: Population Assessment of Tobacco and Health Survey. *Int J Environ Res Public Health*. 2019;16(7):1263. doi:10.3390/ijerph16071263
64. ALHarthi SS, BinShabaib M, Akram Z, Rahman I, Romanos GE, Javed F. Impact of cigarette smoking and vaping on the outcome of full-mouth ultrasonic scaling among patients with gingival inflammation: a prospective study. *Clin Oral Investig*. 2019;23(6):2751-2758. doi:10.1007/s00784-018-2725-2
65. Maloney SF, Breland A, Soule EK, et al. Abuse Liability Assessment of an Electronic Cigarette in Combustible Cigarette Smokers. *Exp Clin Psychopharmacol*. 2019;27(5):443-454. doi:10.1037/pha0000261
66. Franzen KF, Willig J, Cayo Talavera S, et al. E-cigarettes and cigarettes worsen peripheral and central hemodynamics as well as arterial stiffness: A randomized, double-blinded pilot study. *Vasc Med*. 2018;23(5):419-425. doi:10.1177/1358863X18779694
67. Coppeta L, Magrini A, Pietroiusti A, Perrone S, Grana M. Effects of Smoking Electronic Cigarettes on Pulmonary Function and Environmental Parameters. *The Open Public Health Journal*. 2018;11(1). doi:10.2174/1874944501811010360
68. Corbett SE, Nitzberg M, Moses E, et al. Gene Expression Alterations in the Bronchial Epithelium of e-Cigarette Users. *Chest*. 2019;156(4):764-773. doi:10.1016/j.chest.2019.05.022
69. Hamad SH, Brinkman MC, Tsai YH, et al. Pilot Study to Detect Genes Involved in DNA Damage and Cancer in Humans: Potential Biomarkers of Exposure to E-Cigarette Aerosols. *Genes (Basel)*. 2021;12(3):448. doi:10.3390/genes12030448
70. Staudt MR, Salit J, Kaner RJ, Hollmann C, Crystal RG. Altered lung biology of healthy never smokers following acute inhalation of E-cigarettes. *Respiratory Research*. 2018;19(1):78. doi:10.1186/s12931-018-0778-z
71. Mastrangeli S, Carnevale R, Cavarretta E, et al. Predictors of oxidative stress and vascular function in an experimental study of tobacco versus electronic cigarettes: A post hoc analysis of the SUR-VAPES 1 Study. *Tob Induc Dis*. 2018;16:18. doi:10.18332/tid/89975
72. Alasmari F, Crotty Alexander LE, Hammad AM, et al. E-cigarette aerosols containing nicotine modulate nicotinic acetylcholine receptors and astroglial glutamate transporters in mesocorticolimbic brain regions of chronically exposed mice. *Chem Biol Interact*. 2021;333:109308. doi:10.1016/j.cbi.2020.109308
73. Crotty Alexander LE, Drummond CA, Hepokoski M, et al. Chronic inhalation of e-cigarette vapor containing nicotine disrupts airway barrier function and induces systemic inflammation and multiorgan fibrosis in mice. *Am J Physiol Regul Integr Comp Physiol*. 2018;314(6):R834-R847. doi:10.1152/ajpregu.00270.2017
74. Pham K, Huynh D, Le L, et al. E-cigarette promotes breast carcinoma progression and lung metastasis: Macrophage-tumor cells crosstalk and the role of CCL5 and VCAM-1. *Cancer Lett*. 2020;491:132-145. doi:10.1016/j.canlet.2020.08.010
75. Szafran BN, Pinkston R, Perveen Z, et al. Electronic-Cigarette Vehicles and Flavoring Affect Lung Function and Immune Responses in a Murine Model. *Int J Mol Sci*. 2020;21(17):6022. doi:10.3390/ijms21176022

76. Lee WH, Ong SG, Zhou Y, et al. Modeling Cardiovascular Risks of E-Cigarettes With Human-Induced Pluripotent Stem Cell-Derived Endothelial Cells. *J Am Coll Cardiol*. 2019;73(21):2722-2737. doi:10.1016/j.jacc.2019.03.476
77. Vermehren MF, Wiesmann N, Deschner J, Brieger J, Al-Nawas B, Kämmerer PW. Comparative analysis of the impact of e-cigarette vapor and cigarette smoke on human gingival fibroblasts. *Toxicol In Vitro*. 2020;69:105005. doi:10.1016/j.tiv.2020.105005
78. Tommasi S, Caliri AW, Caceres A, et al. Deregulation of Biologically Significant Genes and Associated Molecular Pathways in the Oral Epithelium of Electronic Cigarette Users. *Int J Mol Sci*. 2019;20(3):738. doi:10.3390/ijms20030738
79. Rowell TR, Keating JE, Zorn BT, Glish GL, Shears SB, Tarran R. Flavored e-liquids increase cytoplasmic Ca2+ levels in airway epithelia. *Am J Physiol Lung Cell Mol Physiol*. 2020;318(2):L226-L241. doi:10.1152/ajplung.00123.2019
80. Corey CG, Chang JT, Rostron BL. Electronic nicotine delivery system (ENDS) battery-related burns presenting to US emergency departments, 2016. *Injury Epidemiology*. 2018;5(1):4. doi:10.1186/s40621-018-0135-1
81. Chang JT, Wang B, Chang CM, Ambrose BK. National estimates of poisoning events related to liquid nicotine in young children treated in US hospital emergency departments, 2013–2017. *Injury Epidemiology*. 2019;6(1):10. doi:10.1186/s40621-019-0188-9

Fig 1. PRISMA flow diagram demonstrating selection of studies (the KCL review Validation)

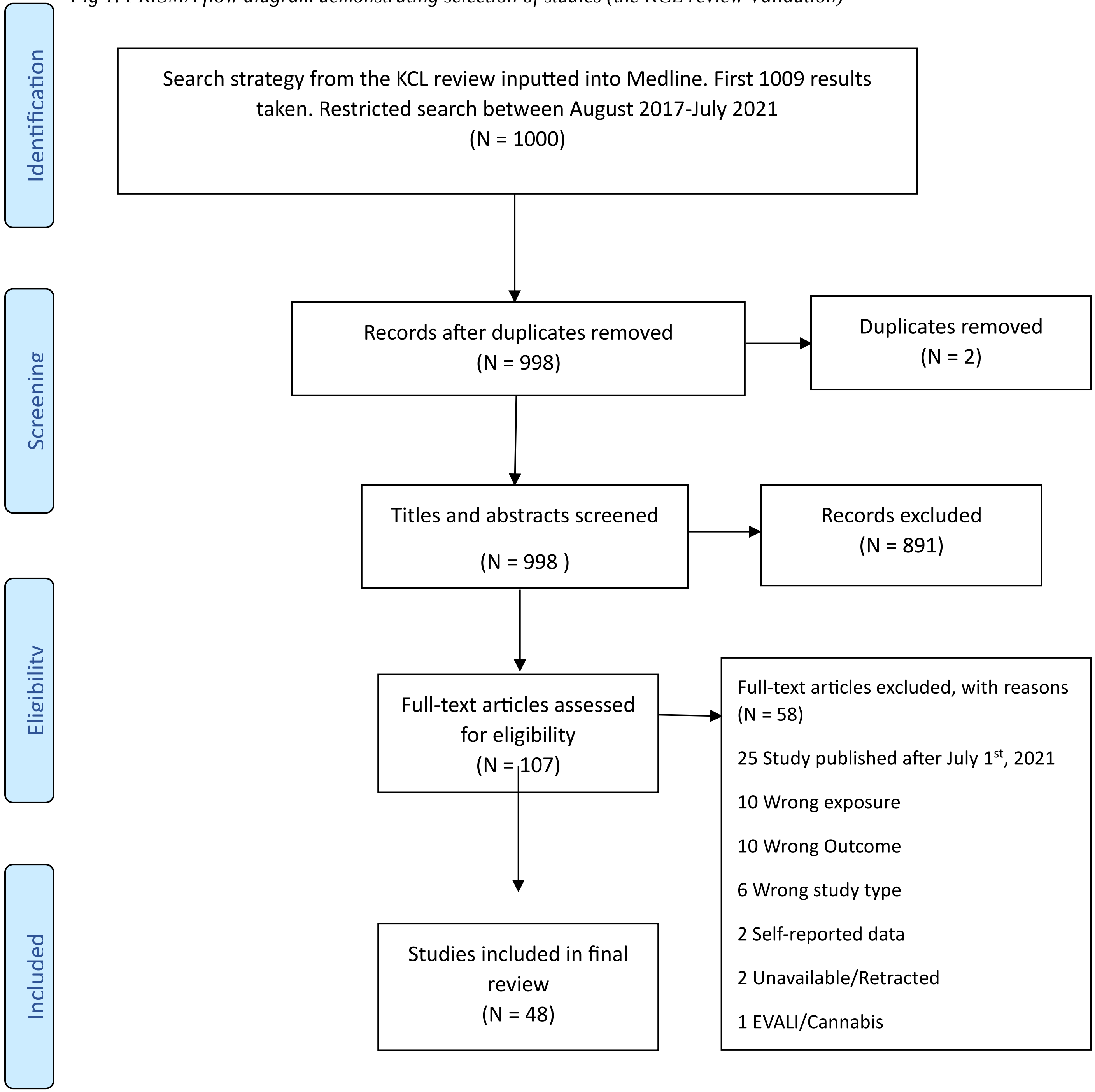

PRISMA Reporting Checklist

Section/topic	#	Checklist item	Reported on page #
**TITLE**			
Title	1	Identify the report as a systematic review, meta-analysis, or both.	1
**ABSTRACT**			
Structured summary	2	Provide a structured summary including, as applicable: background; objectives; data sources; study eligibility criteria, participants, and interventions; study appraisal and synthesis methods; results; limitations; conclusions and implications of key findings; systematic review registration number.	2
**INTRODUCTION**			
Rationale	3	Describe the rationale for the review in the context of what is already known.	3
Objectives	4	Provide an explicit statement of questions being addressed with reference to participants, interventions, comparisons, outcomes, and study design (PICOS).	3,4
**METHODS**			
Protocol and registration	5	Indicate if a review protocol exists, if and where it can be accessed (e.g., Web address), and, if available, provide registration information including registration number.	4
Eligibility criteria	6	Specify study characteristics (e.g., PICOS, length of follow-up) and report characteristics (e.g., years considered, language, publication status) used as criteria for eligibility, giving rationale.	5
Information sources	7	Describe all information sources (e.g., databases with dates of coverage, contact with study authors to identify additional studies) in the search and date last searched.	4
Search	8	Present full electronic search strategy for at least one database, including any limits used, such that it could be repeated.	Supplemental Material 1
Study selection	9	State the process for selecting studies (i.e., screening, eligibility, included in systematic review, and, if applicable, included in the meta-analysis).	5
Data collection process	10	Describe method of data extraction from reports (e.g., piloted forms, independently, in duplicate) and any processes for obtaining and confirming data from investigators.	5,6
Data items	11	List and define all variables for which data were sought (e.g., PICOS, funding sources) and any assumptions and simplifications made.	5,6
Risk of bias in individual studies	12	Describe methods used for assessing risk of bias of individual studies (including specification of whether this was done at the study or outcome level), and how this information is to be used in any data synthesis.	6,7
Summary measures	13	State the principal summary measures (e.g., risk ratio, difference in means).	6
Synthesis of results	14	Describe the methods of handling data and combining results of studies, if done, including measures of consistency (e.g., I^2) for each meta-analysis.	5,6

PRISMA 2009 Checklist

Section/topic	#	Checklist item	Reported on page #
Risk of bias across studies	15	Specify any assessment of risk of bias that may affect the cumulative evidence (e.g., publication bias, selective reporting within studies).	6,7
Additional analyses	16	Describe methods of additional analyses (e.g., sensitivity or subgroup analyses, meta-regression), if done, indicating which were pre-specified.	5
**RESULTS**			
Study selection	17	Give numbers of studies screened, assessed for eligibility, and included in the review, with reasons for exclusions at each stage, ideally with a flow diagram.	8, Fig 1
Study characteristics	18	For each study, present characteristics for which data were extracted (e.g., study size, PICOS, follow-up period) and provide the citations.	8, Table1
Risk of bias within studies	19	Present data on risk of bias of each study and, if available, any outcome level assessment (see item 12).	8,9, Supplemental Material 4
Results of individual studies	20	For all outcomes considered (benefits or harms), present, for each study: (a) simple summary data for each intervention group (b) effect estimates and confidence intervals, ideally with a forest plot.	9-11, Supplemental Material 2-3, Fig 2-4
Synthesis of results	21	Present results of each meta-analysis done, including confidence intervals and measures of consistency.	Not done
Risk of bias across studies	22	Present results of any assessment of risk of bias across studies (see Item 15).	8,9, Supplemental Material 4
Additional analysis	23	Give results of additional analyses, if done (e.g., sensitivity or subgroup analyses, meta-regression [see Item 16]).	12, Supplemental Material 2-3, Fig 4
**DISCUSSION**			
Summary of evidence	24	Summarize the main findings including the strength of evidence for each main outcome; consider their relevance to key groups (e.g., healthcare providers, users, and policy makers).	12-13
Limitations	25	Discuss limitations at study and outcome level (e.g., risk of bias), and at review-level (e.g., incomplete retrieval of identified research, reporting bias).	13-14
Conclusions	26	Provide a general interpretation of the results in the context of other evidence, and implications for future research.	14
**FUNDING**			

Funding	27	Describe sources of funding for the systematic review and other support (e.g., supply of data); role of funders for the systematic review.	Title Page

From: Moher D, Liberati A, Tetzlaff J, Altman DG, The PRISMA Group (2009). Preferred Reporting Items for Systematic Reviews and Meta-Analyses: The PRISMA Statement. PLoS Med 6(7): e1000097. doi:10.1371/journal.pmed1000097

© 2025 Kundu A. et al.
